# Supplementary material for: Detection of cooperatively bound transcription factor pairs using ChIP-seq peak intensities and expectation maximization
Source: PLoS One. 2018 Jul 17;13(7):e0199771. doi: 10.1371/journal.pone.0199771 (PMC6049898; doi:10.1371/journal.pone.0199771)
Supplement: S1 Appendix — (PDF) [file pone.0199771.s001.pdf]

A ChIP-seq pipeline for peak calling and processing knockout data

Peak intensity is a measure of binding affinity, and in terms of the narrowPeak and broadPeak output format of most ChIP-seq peak callers, this could be the signal value (7th column), $-\log_{10}$(p-value) (8th column), or the $-\log_{10}$(q-value) (9th column) of each line in the peak call file. The signal value is typically computed from the number of sequence reads that originate from a bound genomic location. The p-value is computed from the signal value, which is a measure of statistical significance of the peak call. The q-value of each peak call is computed by adjusting the p-value to control the false discovery rate of the peak call set [10], which is a correction for multiple hypothesis testing. A peak call with a larger signal value has a smaller p- and q-value, which indicates that it is more likely to reflect an actual protein-DNA binding event. Thus, a larger signal value will translate to a larger $-\log_{10}$(p-value) and $-\log_{10}$(q-value). Across all datasets, we refer to the signal value (6th column) as the peak intensity of that peak call.

A.1 ChIP-seq of FOXA1, HNF4A, and CEBPA from *M. musculus* liver:

We aligned the raw sequence reads (ArrayExpress, accession number: E-MTAB-1414) from the experiment [1] to the 2007 UCSC mm9 release of the C57/BL6 strain of the mouse genome, using the BWA (v0.7.12) aligner with default settings [4]. We ran MACS2 (v2.1.0) [5], with its default settings, to call peaks on each of these alignments. Peaks were called with a liberal p-value threshold of 10^{-3}. Since the wild-type ChIP-seq data consisted of two biological replicates, we pooled these aligned reads into a single file and called peaks using MACS2. We ran MACS2 with default settings, which discards aligned reads that are PCR duplicates before calling peaks..

We then applied a second criterion to filter peak calls. The use of a relatively liberal p-value threshold of 10^{-3} while calling peaks, and the pooling of aligned reads before calling peaks, was necessary in order to compute the irreproducible discovery rate (IDR) [6, 7] of each peak. We computed the IDR of each peak with the idr script (v2.0) [6], and retained peaks whose IDR was less than 1%. We then ranked peaks according to their MACS2 signal values, with the top ranked peak having the largest signal value. We divided these ranks by the total number of peaks in the ChIP-seq profile to obtain a normalized rank for each peak, which is equivalent to the quantile of that peak intensity within the profile. Significant changes in these peak ranks were used to detect cooperative binding events while comparing peak calls between wild-type and knockout ChIP-seq data.

Because the ChIP-seq of FOXA1 in Δ*HNF4A* and Δ*CEBPA* cells, HNF4A in Δ*CEBPA* cells, and CEBPA in Δ*HNF4A* cells were not performed in replicates, we could not use the IDR criterion to filter peaks. Instead, for these, we filtered peak calls using the q-value of each peak call as computed by MACS2. We retained only those peaks whose q-values were less than 0.01 for further analysis. These peak calls were finally used to detect cooperative binding in FOXA1-HNF4A, FOXA1-CEBPA, HNF4A-CEBPA and CEBPA-HNF4A pairs.

Since we filtered peak calls based on q-values in the other datasets we analyzed, we also created a separate set of peak calls for the ChIP-seq of FOXA1, HNF4A and CEBPA (in wild-type cells), which were filtered according to their q-values instead of their IDR values. This was to ensure that none of our results from this set of data were affected due to IDR being employed to filter peak calls in wild-type cells and q-values being employed to filter peak calls in knockout cells. To create this separate q-value based peak call set for FOXA1, CEBPA and HNF4A, we called peaks on the merged alignments of FOXA1, CEBPA and HNF4A using MACS2 (v2.1.0) using a q-value threshold of 0.05.

A.2 ChIP-seq of GCN4, RTG3 in *S. cerevisiae*:

We aligned raw sequence reads [8] from the ChIP-seq libraries of GCN4, RTG3 (accession Number GSE60281) to the S288C reference genome (R64-2-1) of *S. cerevisiae*, available at the Saccharomyces Genome Database [9].

We followed the same procedure as with the *M. musculus* data, with some changes. ChIP-seq reads from GCN4 and RTG3 were available in three replicates. In these datasets, we merged the read alignments of all three replicates and

called peaks on these combined alignments. We called peaks on this merged set using MACS2, with a p-value threshold of 0.1, and retained peaks whose q-values were less than 0.1. MACS2 was run with the additional `--nomodel --extsize 147 -g 1.21e7` options.

A.3 ChIP-seq of FIS and CRP in *E. coli* from early-exponential (EE) and mid-exponential (ME) phase cultures:

For FIS and CRP ChIP-seq datasets, we utilized pre-computed peak calls that were available on the GEO database with accession number GSE92255. Though the ChIP-seq experiments were carried out in replicates, these peaks were called by running MACS2 on merged alignments of sequence reads from both replicates. The peak calls in this set were filtered such that all peaks had a q-value less than 0.05.

In order to analyze whether these samples were sequenced to saturation, we had to call peaks on individual replicates. We followed the same peak calling protocol for the individual replicates as was stated on the GEO database entry. We used MACS2 (v2.0.1) in paired-end mode (`-f BAMPE`) to call peaks on the invidividual replicates, with those peak calls whose q-value is less than 0.05 being retained. To call peaks on the merged replicates, we passed alignment files of both individual ChIP and input replicates to the `-t` and `-c` options of MACS2 (v2.0.1).

We would like to highlight here that the pipeline used for calling peaks in the individual early-exponential (ΔFIS) CRP ChIP-seq replicates was slightly modified from those of the other datasets, where MACS2 was (v2.0.1) was run with the additional options (`--keep-dup 'all'`). This was in line with the original study that reported this data, where PCR duplicates were kept in the individual replicates because MACS2 reported no peaks in the second ChIP-seq replicate. However, the peak calls for the merged replicates were called with the default PCR duplicate removal options for MACS2.

B Summary of ChIP-seq data analyzed

Genotype	ChIP TF	Replicate	# reads	# aligned reads	# unique aligned reads	# peaks
ΔCEBPA	FOXA1	1	32,597,714	30,701,413	27,118,490	64,953
ΔCEBPA	HNF4A	1	27,398,895	26,079,513	23,383,347	69,075
ΔCEBPA	Input	1	40,186,137	39,232,917	35,379,794	
ΔHNF4A	FOXA1	1	34,759,222	33,201,810	26,601,481	40,269
ΔHNF4A	Input	1	33,549,524	31,203,895	28,893,620	
WT	CEBPA	1	8,920,790	8,169,446	6,890,896	63,524
WT	CEBPA	2	22,741,226	22,372,286	18,922,926	73,781
WT	CEBPA	Merged	31,662,016	30,541,732	25,223,529	85,095
WT	FOXA1	1	20,036,435	17,243,099	14,020,357	90,136
WT	FOXA1	2	22,693,631	22,332,782	19,149,183	95,507
WT	FOXA1	Merged	42,730,066	39,575,881	31,915,168	115,023
WT	HNF4A	1	17,406,988	16,626,822	12,672,074	109,973
WT	HNF4A	2	23,428,817	22,667,764	17,226,195	104,777
WT	HNF4A	Merged	40,83,5805	39,294,586	27,411,711	128,858
WT	Input	1	17,224,577	16,932,945	14,827,244	
WT	Input	2	23,620,903	23,324,876	21,239,295	
WT	Input	Merged	40,845,480	40,257,821	35,879,535	

Table A: Summary of number of reads and peak calls in each ChIP-seq replicate of FOXA1, HNF4A and CEBPA. As stated in A Section, for FOXA1 (ΔCEBPA), FOXA1 (ΔHNF4A) and FOXA1 (ΔHNF4A) datasets, only a single replicate of ChIP-seq was performed. The peak calls for these data sets have a q-value less than 0.05. For the remaining datasets where ChIP-seq was performed in two replicates, the number of peaks shown for each replicate are those whose p-value is less than 0.001. For the merged replicates, those peaks whose IDR values are less than 0.01 are then retained for further analyses.

Genotype	ChIP TF	Replicate	# reads	# aligned reads	# unique aligned reads	# peaks
ΔCRP	FIS	1	23,985,128	23,362,193	5,738,887	1,239
ΔCRP	Input	1	28,139,076	27,388,243	9,647,041	
ΔCRP	FIS	2	13,108,872	11,173,807	3,621,603	1,079
ΔCRP	Input	2	16,455,832	16,013,138	12,482,398	
ΔCRP	FIS	Merged	37,094,000	34,536,000	9,313,430	1,321
ΔCRP	Input	Merged	44,594,908	43,401,381	21,914,390	
ΔFIS	CRP	1	32,920,738	31,313,012	11,823,828	995
ΔFIS	Input	1	24,519,138	24,017,307	12,327,642	
ΔFIS	CRP	2	18,908,968	18,087,796	9,824,367	1,152
ΔFIS	Input	2	22,293,760	21,309,317	1,831,874	
ΔFIS	CRP	Merged	51,829,706	49,400,808	21,286,187	614
ΔFIS	Input	Merged	46,812,898	45,326,624	14,153,427	
WT	CRP	1	19,698,248	18,916,551	5,209,510	1,373
WT	Input	1	19,179,572	18,348,601	11,306,674	
WT	CRP	2	25,513,900	23,569,041	6,775,190	1,347
WT	Input	2	26,487,432	24,506,386	8,768,154	
WT	CRP	Merged	45,212,148	42,485,592	11,915,936	1,551
WT	Input	Merged	45,667,004	42,854,987	19,951,977	
WT	FIS	1	12,387,350	11,731,171	2,589,086	410
WT	Input	1	20,109,602	19,328,277	2,137,289	
WT	FIS	2	10,176,622	9,836,656	1,169,305	807
WT	Input	2	11,337,766	11,094,687	5,186,936	
WT	FIS	Merged	22,563,972	21,567,827	3,752,888	594
WT	Input	Merged	31,447,368	30,422,964	7,316,641	

Table B: Summary of number of paired-end reads in each ChIP and input samples of CRP and FIS for *E. coli* cells in the early exponential (EE) growth phase.

Genotype	ChIP TF	Replicate	# reads	# aligned reads	# unique aligned reads	# peaks
ΔCRP	FIS	1	17,124,050	16,491,466	7,438,622	1,387
ΔCRP	Input	1	36,771,916	35,945,565	32,201,951	
ΔCRP	FIS	2	11,561,622	11,263,776	7,542,848	693
ΔCRP	Input	2	16,416,034	15,936,931	12,770,554	
ΔCRP	FIS	Merged	28,685,672	27,755,242	14,850,235	737
ΔCRP	Input	Merged	53,187,950	51,882,496	44,222,579	
ΔFIS	CRP	1	25,309,640	24,706,744	6,887,892	305
ΔFIS	Input	1	20,997,384	20,696,474	14,398,594	
ΔFIS	CRP	2	27,433,692	26,177,465	11,004,142	313
ΔFIS	Input	2	20,319,816	19,563,144	18,529,135	
ΔFIS	CRP	Merged	52,743,332	50,884,209	17,801,841	175
ΔFIS	Input	Merged	41,317,200	40,259,618	32,537,398	
WT	CRP	1	21,544,318	20,039,437	2,570,734	442
WT	Input	1	14,314,998	13,239,455	2,127,070	
WT	CRP	2	8,777,264	8,327,033	967,632	387
WT	Input	2	19,743,696	17,831,092	4,203,300	
WT	CRP	Merged	30,321,582	28,366,470	3,537,937	578
WT	Input	Merged	34,058,694	31,070,547	6,328,296	
WT	FIS	1	18,113,890	17,658,051	1,355,076	1,367
WT	Input	1	14,344,636	14,038,616	7,880,300	
WT	FIS	2	11,837,996	11,586,429	5,538,472	1,451
WT	Input	2	16,098,716	15,885,657	6,515,651	
WT	FIS	Merged	29,951,886	29,244,480	6,885,629	1,545
WT	Input	Merged	30,443,352	29,924,273	14,324,522	

Table C: Summary of number of reads in each ChIP and input samples of CRP and FIS for *E. coli* cells in the mid-exponential (ME) growth phase.

Genotype	ChIP TF	Replicate	# reads	# aligned reads	# unique aligned reads	# peaks
ΔGCN4	RTG3	1	8,235,710	2,612,756	1,962,795	934
ΔGCN4	Input	1	5,900,814	5,536,176	3,697,208	
ΔGCN4	RTG3	2	7,542,821	5,544,101	4,008,363	1,028
ΔGCN4	Input	2	5,813,106	5,509,474	4,041,401	
ΔGCN4	RTG3	3	7,463,361	4,443,591	2,951,557	1,911
ΔGCN4	Input	3	5,456,967	4,970,647	3,324,457	
ΔGCN4	RTG3	Merged	23,241,892	12,600,448	7,510,405	1,083
ΔGCN4	Input	Merged	17,170,887	16,016,297	8,748,603	
ΔRTG3	GCN4	1	5,909,379	399,292	367,937	3,574
ΔRTG3	Input	1	4,242,803	3,817,565	2,592,976	
ΔRTG3	GCN4	2	5,378,351	3,949,729	2,951,575	2,907
ΔRTG3	Input	2	3,803,482	3,446,105	2,396,890	
ΔRTG3	GCN4	3	7,424,526	3,446,601	2,456,501	4,260
ΔRTG3	Input	3	4,333,487	3,819,966	2,651,921	
ΔRTG3	GCN4	Merged	18,712,256	7,795,622	5,072,285	4,463
ΔRTG3	Input	Merged	12,379,772	11,083,636	6,355,054	
WT	GCN4	1	6,273,651	3,390,654	2,368,027	4,336
WT	Input	1	4,785,870	4,267,874	2,725,285	
WT	GCN4	2	4,074,996	2,772,747	2,171,662	3,909
WT	Input	2	3,173,721	2,857,929	2,010,610	
WT	GCN4	3	8,668,159	6,150,987	4,174,531	3,716
WT	Input	3	3,901,564	3,493,325	2,365,151	
WT	GCN4	Merged	19,016,806	12,314,388	7,236,323	5,165
WT	Input	Merged	11,861,155	10,619,128	5,949,413	
WT	RTG3	1	7,062,903	3,086,243	2,065,954	4,272
WT	Input	1	4,079,500	3,699,735	2619074	
WT	RTG3	2	5,568,366	3,350,249	2,469,440	2,850
WT	Input	2	4,962,298	4,531,299	3,170,068	
WT	RTG3	3	6,628,678	2,278,979	1,691,413	3,659
WT	Input	3	2,999,374	2,762,809	2,079,424	
WT	RTG3	Merged	19,259,947	8,715,471	5,400,260	4,206
WT	Input	Merged	12,041,172	10,993,843	6,715,409	

Table D: Summary of number of reads and peaks in each ChIP and input samples of GCN4 and RTG3. The number of peaks shown in the table for each replicate (and the merged alignments) are those whose p-values are less than 0.1. The final peaks that were used for the remaining analyses were called on the merged alignments, and had a q-value less than 0.1. See A Section for details on how the replicates were chosen.

Data set	n_{double} (n_{peaks}, f)	Losses	Increases	Decreases	Unchanged	Cooperative
FOXA1-HNF4A	19858/30428 (0.65)	7224/9606 (0.75)	1025/2178 (0.47)	1469/1933 (0.76)	10140/16711 (0.61)	8249/19858
FOXA1-CEBPA	12063/30428 (0.4)	1281/2358 (0.54)	452/1846 (0.24)	877/1589 (0.55)	9453/24635 (0.38)	1733/12063
HNF4A-CEBPA	13730/39955 (0.34)	1667/4219 (0.4)	644/2546 (0.25)	896/1831 (0.49)	10523/31359 (0.34)	2311/13730
(EE) CRP-FIS	487/594 (0.82)	36/70 (0.51)	—	—	451/524 (0.86)	36/487
(EE) FIS-CRP	293/1545 (0.19)	140/935 (0.15)	—	—	153/610 (0.25)	140/293
(ME) CRP-FIS	460/1551 (0.3)	176/1050 (0.17)	—	—	284/501 (0.57)	176/460
GCN4-RTG3	2282/4497 (0.51)	533/1672 (0.32)	—	—	1749/2825 (0.62)	533/1672
RTG3-GCN4	2051/3143 (0.65)	1527/2489 (0.61)	—	—	524/654 (0.8)	1527/2489

Table E: Summary of ChIP-seq data from datasets analyzed in Figure 2. The second column describes the total number of doubly bound regions in each dataset. In parentheses, the total number of target TF peaks, and the fraction of target TF peak regions that are doubly bound are shown ($f = n_{double}/n_{peaks}$). The remaining columns list the number of target TF peaks that were lost, increased in rank, decreased in rank, or unchanged in rank, from genomic regions bound by both target and partner TFs. The numbers in parentheses are the number of target TF peaks that were lost, increased in rank, decreased in rank, or unchanged in rank, from all genomic regions bound by the target TF. The last column is the total fraction of cooperatively bound peaks in each dataset. Statistical tests to detect significant peak rank changes could be carried out only in FOXA1-HNF4A, FOXA1-CEBPA and HNF4A-CEBPA datasets (see A1 and A3 sections).

Data set	n_{double} (n_{peaks}, f)	Losses	Increases	Decreases	Unchanged
FOXA1-HNF4A	6199/10651 (0.58)	1528/2342 (0.65)	347/856 (0.41)	563/759 (0.74)	3761/6694 (0.56)
FOXA1-CEBPA	3827/10651 (0.36)	236/541 (0.44)	158/684 (0.23)	303/554 (0.55)	3130/8872 (0.35)
HNF4A-CEBPA	6991/22650 (0.31)	515/1837 (0.28)	330/1473 (0.22)	513/1097 (0.47)	5633/18243 (0.31)
(EE) CRP-FIS	219/264 (0.83)	4/4 (1.0)	—	—	215/260 (0.83)
(EE) FIS-CRP	131/836 (0.16)	46/437 (0.11)	—	—	85/399 (0.21)
(ME) CRP-FIS	264/585 (0.45)	87/328 (0.27)	—	—	177/257 (0.69)
GCN4-RTG3	380/797 (0.48)	38/125 (0.3)	—	—	342/672 (0.51)
RTG3-GCN4	200/277 (0.72)	128/191 (0.67)	—	—	72/86 (0.84)

Table F: Summary of ChIP-seq data from datasets analyzed in Figure I after indirectly bound peaks have been removed. The second column describes the total number of doubly bound regions in each dataset. In parentheses, the total number of target TF peaks, and the fraction of target TF peak regions that are doubly bound are shown ($f = n_{double}/n_{peaks}$). The remaining columns list the number of target TF peaks that were lost, increased in rank, decreased in rank, or unchanged in rank, from genomic regions bound by both target and partner TFs. The numbers in parentheses are the number of target TF peaks that were lost, increased in rank, decreased in rank, or unchanged in rank, from all genomic regions bound by the target TF. Statistical tests to detect significant peak rank changes could be carried out only in FOXA1-HNF4A, FOXA1-CEBPA and HNF4A-CEBPA datasets (see A1 and A3 sections).

C Estimation of average mappability of *M. musculus*, *S. cerevisiae* and *E. coli* genomes

The single-end reads in the *M. musculus* data we employed was 36 bp in length. We downloaded mappability data for the mm9 mouse genome assembly for 36 bp reads from the UCSC genome browser mm9 download section, which was in the bigWig format. We converted this into a bedgraph file using the bigWigToBedGraph program (version dated 03 April 2018).

For *E. coli* and *S. cerevisiae* genomes, we created mappability files in the wiggle format using the program GEM (v2.5.2.1) [25]. We used GEM to compute the mappability of 42 bp long reads in the *S. cerevisiae* genome and 101 bp long reads in the *E. coli* genome using the `gem-indexer`. These were the read lengths employed in the original studies from which we sourced the ChIP-seq data. The sequence of commands used to create the *S. cerevisiae* genome mappability data for 42 bp reads were —

```
gem-indexer -T 4 -c dna -i genome.yeast.fasta -o genome.yeast.index
gem-mappability -T 8 -I genome.yeast.gem -l 42 -o genome.yeast_42
gem-2-wig -I genome.yeast.gem -i genome.yeast_42.mappability -o genome.yeast_42
wig2bed < genome.yeast_42.wig > genome.yeast_42.bedgraph
```

The commands used to create the *E. coli* genome mappability data for 101 bp reads were —

```
gem-indexer -T 4 -c dna -i genome.ecoli.fasta -o genome.ecoli.index
gem-mappability -T 8 -I genome.ecoli.gem -l 101 -o genome.ecoli_101
gem-2-wig -I genome.ecoli.gem -i genome.ecoli_101.mappability -o genome.ecoli_101
wig2bed < genome.ecoli_101.wig > genome.ecoli_101.bedgraph
```

We note that this *E. coli* mappability track was created assuming that the reads were single-end reads. Given that the data we used employed paired-end reads, the mappability of these regions are higher in practice than that calculated by `gem`. The `wig2bed` program (v2.4.32) was part of the BEDOPS suite [26].

From these mappability tracks, we followed the procedure outlined in [24] to compute the mappability of ChIP-seq peaks. For a peak of length L base pairs in length, if the mappability score of a M base pair read at each of the L positions is $m_1, m_2, \ldots, m_L$, then, the average mappability of the peak is computed as $\bar{m} = \sum_{i=1}^{L} m_i/L$. This calculation was performed for all peaks in each peak call file using the `map` program (with options `-o mean -c 4`, where the 4th column of the bedgraph is the mappability score) of the BEDTools suite (v2.25.0).

D Intensities of trimmed peaks of cooperatively bound target TFs are weaker than those of non-cooperatively bound target TFs

Since we constructed target and partner TF peak pair sets by choosing those peak pairs whose peak regions overlapped by at least one base pair, we checked if our results were an artefact of this overlap criterion. When we trimmed all ChIP-seq peaks across datasets to 50 base pairs on either side of their summits, we still found cooperatively bound target TF peaks to be more weakly bound than non-cooperatively bound target TF peaks (D Fig). However, this trend was no longer statistically significant in *S. cerevisiae* and *E. coli* datasets. However, the fact that the notches of the boxplots are larger than the $25-th$ and $75-th$ percentiles of the peak intensity distributions indicates that the number of peaks available in these datasets is small. Thus, the lack of statistical significance of the trends in intensities of these datasets is likely a signature of the small number of peaks.

E Performance of different variants of the CPI-EM algorithm

When CPI-EM was run to compute cooperative binding probabilities in the main text, Lognormal shapes were fitted to the joint probability function of observing cooperative and non-cooperatively bound peak intensity pairs (step 2 of the CPI-EM algorithm). In Figure L, we compare the detection performance of this version of the CPI-EM algorithm with two other variants that fit Gamma and Gaussian shapes instead of a Lognormal shape. Figure L shows the auROC of these three variants of the CPI-EM algorithm after they were run on all the datasets shown in Figure 1 in the main text. We also compared the auROC of these CPI-EM variants to the peak distance detector, which is described in the Materials and Methods of the main text, and a detector based purely on chance. The chance detector is based on using tosses from a biased coin, whose probability of showing heads is α, to detect cooperative interactions. The area under the ROC of this detector will be 0.5 for any dataset (see J Section). An auROC of 0.5 thus represents the minimum level of detection performance that an algorithm should obtain to be considered a useful detector in practice.

In Figure L, it can be seen that the Log-normal CPI-EM variant has an auROC of at least 0.7, and thus can consistently detect cooperative interactions across all datasets. The Gamma and Gaussian variants, on the other hand, perform close to the level of the chance detector in FOXA1-CEBPA, HNF4A-CEBPA and early-exponential phase CRP-FIS datasets. There is considerable variation in the auROC of the peak distance based algorithm: less than 0.5 in early-exponential phase FIS-CRP and RTG3-GCN4 datasets, but higher than 0.5 in the remaining datasets. The fact that this algorithm can perform worse than a chance detector shows that peak distance, by itself, is an unreliable criterion for detecting cooperative binding. The complete ROC curves of each of the CPI-EM and peak distance algorithms for the datasets in Figure 1 are shown in Figure P.

We checked if the performance of CPI-EM was dependent on instances of indirect binding between target and partner TF peaks and the size of the ChIP-seq peaks input to it. We found that removing indirectly bound peaks of target and partner TFs (Figure QA) did not noticeably alter the auROCs of any of the CPI-EM variants, with the log-normal CPI-EM variant continuing to perform better than chance across all datasets. When we trimmed the peaks of both TFs to within 50 base pair on either side of the peak summits before passing them as inputs to CPI-EM, there was no noticeable drop in the auROCs of any of the CPI-EM variants, except in the case of the mid-exponential phase CRP-FIS dataset. The auROC of the log-normal CPI-EM variant (Figure QB) on this dataset dropped below 0.5.

F Analyzing the extent of saturation of sequencing depth for ChIP-seq datasets

To determine the extent to which the ChIP and input samples were sequenced, we called peaks on ChIP and input samples after sub-sampling 20%,40%,60% and 80% of reads from each file. In samples that have been sequenced to saturation, a small change in the number of reads should not result in a change in the number of peaks called.

We sub-sampled reads from the BAM alignments using the `samtools view -bs` program. This was done by passing values of the form $xy.f$ to the $-s$ flag, where xy was a integer sampled uniformly between 0 and 99, and f was either 2,4,6 or 8, which corresponded to sampling 20,40,60 or 80% of the reads. We ran this command three times for each ChIP and input BAM alignment pair, thus generating three replicates for each sub-sampling fraction. We called peaks on each matched pair of sub-sampled ChIP and input BAM files using the appropriate pipelines described in A Section.

The results of this analysis are shown in K Fig.

G Detecting significant rank changes of target TF peaks after the partner TF is knocked out

To determine if a change in the peak rank of X upon the knocking out of Y is statistically significant, we construct a null distribution that captures the magnitude of rank changes of X expected purely due to variability in the ChIP-seq protocol. Suppose $r_1^{(1)}, r_2^{(1)}, \ldots, r_n^{(1)}$ and $r_1^{(2)}, r_2^{(2)}, \ldots, r_n^{(2)}$ represent the normalized ranks (whose values are between 0 and 1) of n overlapping peaks in biological replicates 1 and 2 of the ChIP-seq of X (in the presence of Y). We then divide the interval $[0, 1]$ into 10 equally sized bins (we verified that changing the number of bins did not drastically change the results), and compute the null rank change probability density $g^k_{null}(x)$ of the $k-th$ bin from the samples $S_k = \{|r_1^{(1)} - r_1^{(2)}|, |r_2^{(1)} - r_2^{(2)}|, \ldots, |r_l^{(1)} - r_l^{(2)}|\}$, where each of $\{r_i^{(1)}\}_{i=1}^n$ falls in the kth bin. X Gaussian kernel density estimator implemented in the Scipy library was used to compute $g^k_{null}(x)$ for each bin. This represents the probability of observing a rank change purely due to inter-replicate variation, conditioned on the bin to which the peak's rank in replicate 1 belongs. The process of computing rank changes separately within each bin better captured the skew expected in rank changes arising from replicate variation. For instance, a peak of X, whose rank in replicate 1 is low, is far more likely to have a higher rank in replicate 2, than a peak with a high rank in replicate 1.

We then proceed to compute the significance of rank changes observed in peaks of X after Y has been knocked out. For this, we computed the ranks $r_1^{(m)}, r_2^{(m)}, \ldots, r_q^{(m)}$ from peaks of X that have been called from merging the read alignments of replicates 1 and 2. The average change in peak rank due to the merging of alignments was close to zero, i.e., the ranks $r_1^{(m)}, r_2^{(m)}, \ldots, r_p^{(m)}$, did not change on average compared to $r_1^{(1)}, r_2^{(1)}, \ldots, r_p^{(1)}$ and $r_1^{(2)}, r_2^{(2)}, \ldots, r_p^{(2)}$ (data not shown), where p is the number of peaks common between peak calls in the replicates and merged alignments. We also compute the ranks $r_1^{\Delta}, r_2^{\Delta}, \ldots, r_q^{\Delta}$ of peak calls from the ChIP-seq of X after Y is knocked out. We then construct the set of rank changes $\{|r_1^{(m)} - r_1^{\Delta}|, (|r_2^{(m)} - r_2^{\Delta}|), \ldots, |r_q^{(m)} - r_q^{\Delta}|\}$. For each rank change, we calculate $p_i = g^k_{null}(|r_i^{(m)} - r_i^{\Delta}|)$, where k is the bin into which $r_i^{(m)}$ falls. This is the probability of observing a rank change of magnitude $|r_i^{(m)} - r_i^{\Delta}|$ purely due to inter-replicate variation, given that $r_i^{(m)}$ belongs to the $k-th$ bin. We finally obtain a sequence of probabilities $p_1, p_2, \ldots, p_q$ corresponding to each rank change observed upon knocking out Y.

We then conduct q one-sided hypothesis tests, each of which test the null hypothesis $H_i : |r_i^{(m)} - r_i^{\Delta}| = 0$. We carry out the hypothesis tests by checking if each $p_i < \alpha$, where α is chosen according to the Benjamini-Hochberg multiple hypothesis testing procedure [10] that sets the false discovery rate at 0.01. Statistics on the number of significant peak rank changes we observed in different datasets are shown in Table E.

We used this procedure to detect significant rank changes in FOXA1-HNF4A, FOXA1-CEBPA, and HNF4A-CEBPA datasets only. This was because (a) the ChIP-seq of FOXA1, HNF4A and CEBPA were carried out in replicates, and (b) the number of peaks that remained after IDR-based filtering was large. This gave us a sufficient number of peaks with which to reliably compute the null rank change distribution. This was not the case in the RTG3-GCN4 and GCN4-RTG3 datasets, where the number of ChIP-seq peaks in the merged alignments (89 in RTG3-GCN4 and 343 in GCN4-RTG3 datasets) was small. We could not detect rank changes in the CRP-FIS and FIS-CRP datasets because peak calls from individual replicates were not available, and hence, the null rank change distributions could not be computed.

H CPI-EM : Estimating parameters required to compute the probability of cooperative binding at a location

The input to the CPI-EM algorithm consists of a set of peak intensity pairs $\mathbf{D} = \{(x_i, y_i)\}_{i=1}^N$, where $\{x_i\}$ and $\{y_i\}$ are peak intensities of the target TF X and partner TF Y. We assume that the joint probability density of peak intensities from all these regions, $p(x, y)$, is a mixture (i.e., a sum) of two densities representing cooperative and non-cooperative peak intensity distributions:

$$p(x, y) = \pi_0 p_0(x, y; \boldsymbol{\theta}_0) + \pi_1 p_1(x, y; \boldsymbol{\theta}_1), \tag{1}$$

where p_0 and p_1 are the joint densities of peak intensities from non-cooperatively and cooperatively bound regions, respectively. $\boldsymbol{\theta}_0$ and $\boldsymbol{\theta}_1$ represent the parameters of both joint distributions. As shown in the main text, we make three assumptions in the CPI-EM algorithm, which we describe and justify in detail below –

- **Assumption 1 :** We assume that $p_0(x, y; \boldsymbol{\theta}_0) = p_0^X(x; \boldsymbol{\theta}_0^X)p_0^Y(y; \boldsymbol{\theta}_0^Y)$ and $p_1(x, y; \boldsymbol{\theta}_1) = p_1^X(x; \boldsymbol{\theta}_0^X)p_1^Y(y; \boldsymbol{\theta}_1^Y)$, where p_0^X,p_0^Y are marginal distributions of $p_0(x, y)$ and p_1^X,$p_1^Y(y)$ are marginal distributions of $p_1(x, y)$. The parameter vectors of the joint and marginal distributions are related as $\boldsymbol{\theta}_0 = (\boldsymbol{\theta}_0^X, \boldsymbol{\theta}_0^Y)$ and $\boldsymbol{\theta}_1 = (\boldsymbol{\theta}_1^X, \boldsymbol{\theta}_1^Y)$. This assumption reduces equation (1) to

$$p(x, y) = \pi_0 p_0^X(x; \boldsymbol{\theta}_0^X)p_0^Y(y; \boldsymbol{\theta}_0^Y) + \pi_1 p_1^X(x; \boldsymbol{\theta}_1^X)p_1^Y(y; \boldsymbol{\theta}_1^Y). \tag{2}$$

  We found this to be a reasonable assumption across all our data sets when we calculated the mutual information (MI) [13] between peak intensities of cooperatively and non-cooperatively bound peak pairs, as determined by partner TF knockouts, across all our data sets (Table G). Mutual information, measured in bits, is a robust measure of statistical dependence between two random variables, whose value is zero if the variables are statistically independent [12].

Data set	(I) Peak Intensity–Peak Intensity			(II) Motif Score–Motif Score		
	(a) All pairs	(b) Coop pairs	(c) Non-coop pairs	(a) All pairs	(b) Coop pairs	(c) Non-coop pairs
**FOXA1-HNF4A**						
Full data	0.02	0.02	0.04	0.02	0.03	0.04
Indirect removed	0.05	0.03	0.07	0.03	0.02	0.10
**FOXA1-CEBPA**						
Full data	0.03	0.02	0.04	0.03	0.02	0.04
Indirect removed	0.06	0.04	0.06	0.02	-0.07	0.02
**HNF4A-CEBPA**						
Full data	0.03	0.04	0.03	0.03	0.03	0.03
Indirect removed	0.05	0.03	0.05	0.04	0.15	0.04
**(EE) CRP-FIS**						
Full data	0.05	-0.02	0.05	0.05	-0.02	0.05
Indirect removed	0.06	0.08	0.05	-0.03	0.12	0.02
**(ME) CRP-FIS**						
Full data	0.02	0.04	0.03	0.01	0.04	0.02
Indirect removed	-0.05	—	—	-0.08	—	—
**GCN4-RTG3**						
Full data	0.05	-0.01	0.045	0.006	0.00	0.10
Indirect removed	—	—	—	—	—	—

Table G: A summary of the MI values estimated between pairs of peak intensities on the one hand and motif scores from the sequences underlying these peaks. We computed the MI separately for cooperatively, non-cooperatively and all doubly bound regions. The MI between target and partner TF peak intensities from both cooperatively and non-cooperatively bound regions are close to zero across all data sets. This is the case even after indirectly bound peaks are removed or if the MI is computed between motif score pairs instead of peak intensity pairs. MI is a non-negative quantity but the estimator we employ can give negative estimates of MI if the true MI in the data is low or if the number of peak pairs available is low. In cases where the number of peak pairs was far too low (typically, < 20), the MI could not be estimated and we have displayed these values as "—".

- **Assumption 2 :** We choose $p_0^X, p_0^Y, p_1^X, p_1^Y$ to be either a Lognormal, Gamma or Gaussian density function, whose expressions and corresponding parameter sets are –

$$\begin{aligned}
\text{Lognormal: } p(x; m, \sigma) &= \frac{e^{-(\ln(x)/m)^2/2(\sigma)^2}}{x\sigma\sqrt{2\pi}} \qquad x \geq 0; m, \sigma > 0 \qquad \boldsymbol{\theta} = (m, \sigma), \\
\text{Gamma: } p(x; \gamma, \beta) &= \frac{(\frac{x}{\beta})^{\gamma-1} \exp\left(-\frac{x}{\beta}\right)}{\beta\Gamma(\gamma)} \qquad x \geq 0; \gamma, \beta > 0 \qquad \boldsymbol{\theta} = (\beta, \gamma), \\
\text{Gaussian: } p(x; \mu, \sigma) &= \frac{\exp\left(-(x-\mu)^2/2\sigma^2\right)}{\sigma\sqrt{2\pi}} \qquad \sigma > 0 \qquad \boldsymbol{\theta} = (\mu, \sigma).
\end{aligned} \tag{3}$$

Across most datasets, we found that the Lognormal distribution tended to best fit peak intensity distributions. This could be seen in terms of the log-likelihood scores obtained from fitting the three distributions individually to peak intensities of target and partner TFs from cooperatively and non-cooperatively bound regions. The log-likelihood score obtained from fitting these distributions to a set of peak intensities of a given TF is computed as

$$\log\left(P(\mathbf{Z}|\boldsymbol{\Theta})\right) = \sum_{i=1}^{N} \log\left(p(z_i; \boldsymbol{\Theta})\right)$$

where p is either the cooperative (p_1) or non-cooperative (p_0) density. $\mathbf{Z}$ is $\{x_i\}_{i=1}^N$ or $\{y_i\}_{i=1}^N$, which are target or partner TF peak intensities, respectively. $\boldsymbol{\Theta}$ are parameters of the distribution chosen for p. A larger log-likelihood value indicates a better fit to data.

We computed $\boldsymbol{\Theta}$ for each distribution using maximum likelihood estimates of these parameters. We used `fit` routines of the `stats` library of the Python package SciPy [11] to compute these estimates. The log-likelihood values calculated for each of the three distributions across all ChIP-seq datasets is shown in Table H.

Dataset	Cooperative			Non-cooperative		
**Target TF**	Cooperative ($p_1^X(x)$)			Non-cooperative ($p_0^X(x)$)		
	Lognormal	Gamma	Gaussian	Lognormal	Gamma	Gaussian
FOXA1-HNF4A	**−37367**	−37694	−39881	**−148661**	−150528	−164422
FOXA1-CEBPA	**−7413**	−7497	−8032	**−125906**	−125971	−134171
HNF4A-CEBPA	**−10360**	−10419	−10968	−145841	**−144745**	−149395
(EE) FIS-CRP	−82	**−81**	−88	**−1756**	−4064	−2063
(EE) CRP-FIS	−567	**−565**	−576	−1145	**−1144**	−1150
(ME) FIS-CRP	**−455**	−457	−518	−620	**−619**	−645
GCN4-RTG3	286	**287**	239	290	**300**	45
RTG3-GCN4	1203	**1221**	945	193	**198**	124
**Partner TF**	Cooperative ($p_1^Y(y)$)			Non-cooperative ($p_0^Y(y)$)		
	Lognormal	Gamma	Gaussian	Lognormal	Gamma	Gaussian
FOXA1-HNF4A	−46926	**−46736**	−47168	**−162580**	−162688	−170887
FOXA1-CEBPA	−9556	**−9546**	−9793	**−123278**	−123870	−132830
HNF4A-CEBPA	**−11709**	−11710	−12085	**−128582**	−129452	−139457
(EE) FIS-CRP	−124	**−114**	−128	−1640	**−1636**	−1661
(EE) CRP-FIS	**−656**	−1329	−801	**−1199**	−2699	−1402
(ME) FIS-CRP	−546	**−542**	−642	**−621**	−1127	−713
GCN4-RTG3	**424**	196	334	**956**	36	673
RTG3-GCN4	**459**	−214	169	49	**56**	−11

Table H: Log-likelihood values obtained from fitting log-normal, Gaussian and Gamma distributions to cooperative and non-cooperative peak intensities of the datasets shown in Table E. The maximum log-likelihood values are indicated in bold. Across most datasets, the log-normal distribution typically provides the best fit to peak intensity distributions.

- **Assumption 3 :** A cooperatively bound target TF is, on average, more weakly bound than a non-cooperatively bound target TF. This implies that $\langle p_1^X(x)\rangle < \langle p_0^X(x)\rangle$. We found this to be a reasonable assumption since in Figure

2 in the main text, cooperatively bound target TF peak intensities were significantly lower than non-cooperatively bound target TF peak intensities across all datasets.

From equation (3) each of $\boldsymbol{\theta}_0^X, \boldsymbol{\theta}_1^X, \boldsymbol{\theta}_0^Y, \boldsymbol{\theta}_1^Y$ consist of two parameters, irrespective of whether p_1 is a Lognormal, Gamma or Gaussian density function. Along with π_0, there are thus a total of 9 parameters that we need to estimate from $\mathbf{D}$ in order to compute the probability of each peak intensity pair in $\mathbf{D}$ being cooperative –

$$p_i^{coop} \equiv P(L_i = 1 | x_i, y_i) = \frac{\pi_1 p_1(x_i, y_i; \boldsymbol{\theta}_1)}{\pi_1 p_1(x_i, y_i; \boldsymbol{\theta}_0) + \pi_0 p_0(x_i, y_i; \boldsymbol{\theta}_0).} \tag{4}$$

$$= \frac{\pi_1 p_1^X(x_i; \boldsymbol{\theta}_1^X) p_1^Y(y_i; \boldsymbol{\theta}_1^Y)}{\pi_0 p_0^X(x_i; \boldsymbol{\theta}_0^X) p_0^Y(y_i; \boldsymbol{\theta}_0^Y) + \pi_1 p_1^X(x_i; \boldsymbol{\theta}_1^X) p_1^Y(y_i; \boldsymbol{\theta}_1^Y)}. \tag{5}$$

H.1 The expectation-maximization (EM) algorithm

We use the expectation-maximization (EM) algorithm [14, 15] to estimate the parameters in equations (5) and (2). The output of the EM algorithm is a single set of parameters $\boldsymbol{\Theta} = (\pi_0, \boldsymbol{\theta}_0^X, \boldsymbol{\theta}_0^Y, \boldsymbol{\theta}_1^X, \boldsymbol{\theta}_1^Y)$ that maximizes the log-likelihood $\log P(\mathbf{D}, \mathbf{L} | \boldsymbol{\Theta})$, where $\mathbf{D}$ represents the peak intensity pairs $\{(x_i, y_i)\}_{i=1}^N$ and $\mathbf{L} = (L_1, L_2, \ldots, L_N)$ are labels assigned to each of the N locations, where $L_i = 1$ represents cooperative binding and $L_i = 0$ represents non-cooperative binding.

The expectation-maximization algorithm [14, 15] does this by computing a function $Q(\boldsymbol{\Theta}, \boldsymbol{\Theta}')$, which is the expected value of the log-likelihood $\log P(\mathbf{D}, \mathbf{L} | \boldsymbol{\Theta})$, given an earlier estimate of $\boldsymbol{\Theta} = \boldsymbol{\Theta}'$ [16]:

$$Q(\boldsymbol{\Theta}, \boldsymbol{\Theta}') = \sum_{\mathbf{L} \in S} \log \big(P(\mathbf{D}, \mathbf{L} | \boldsymbol{\Theta}) \big) P(\mathbf{L} | \mathbf{D}, \boldsymbol{\Theta}'), \tag{6}$$

where S represents the set of all possible values of L.

Briefly, the EM algorithm starts with an initial guess $\boldsymbol{\Theta}^{(0)}$, and computes a value $\boldsymbol{\Theta}^{(1)}$ such that $Q(\boldsymbol{\Theta}, \boldsymbol{\Theta}^{(0)})$ is maximized with respect to $\boldsymbol{\Theta}$, where $\boldsymbol{\Theta}^{(0)}$ is kept constant. EM then computes $\boldsymbol{\Theta}^{(2)}$ in the next iteration to maximize $Q(\boldsymbol{\Theta}, \boldsymbol{\Theta}^{(1)})$ with respect to $\boldsymbol{\Theta}$, where $\boldsymbol{\Theta}^{(1)}$ is kept constant. This iteration increases the value of Q, i.e., $Q(\boldsymbol{\Theta}^{(2)}, \boldsymbol{\Theta}^{(1)}) > Q(\boldsymbol{\Theta}^{(1)}, \boldsymbol{\Theta}^{(0)}$. Thus, one run of the EM procedure generates a sequence of values $\boldsymbol{\Theta}^{(0)}, \boldsymbol{\Theta}^{(1)}, \boldsymbol{\Theta}^{(2)}, \ldots, \boldsymbol{\Theta}^{(n)}$ which can be proven [14] to satisfy $Q(\boldsymbol{\Theta}^{(1)}, \boldsymbol{\Theta}^{(0)}) \leq Q(\boldsymbol{\Theta}^{(2)}, \boldsymbol{\Theta}^{(1)}) \leq \ldots Q(\boldsymbol{\Theta}^{(n)}, \boldsymbol{\Theta}^{(n-1)})$. EM terminates, say, at the $n-th$ iteration, when Q converges to a local maximum. This local maximum is guaranteed to be a local maximum of $\log P(\mathbf{D}, \mathbf{L} | \boldsymbol{\Theta})$ [16]. $\boldsymbol{\Theta}^{(n)}$ is then substituted in equation (5) to compute the probability of each peak intensity pair being labeled cooperative.

We now describe the Q function employed in CPI-EM, and the implementation of the EM iteration process in detail below.

The set S of all possible labels $\mathbf{L}$ in equation (6) consists of 2^N elements because each element of $\mathbf{L}$ takes on values of either 0 or 1. This is a very large number of terms that need to be added to evaluate Q. However, Q simplifies to a sum over N terms for our model of cooperative binding. Q can be rewritten as

$$Q(\boldsymbol{\Theta}, \boldsymbol{\Theta}') = \sum_{\mathbf{L} \in S} \log \big(P(\mathbf{D}, \mathbf{L} | \boldsymbol{\Theta}) \big) P(\mathbf{L} | \mathbf{D}, \boldsymbol{\Theta}') = \sum_{\mathbf{L} \in S} \log \big(P(\mathbf{L} | \mathbf{D}, \boldsymbol{\Theta}) \, P(\mathbf{D} | \boldsymbol{\Theta}) \big) P(\mathbf{L} | \mathbf{D}, \boldsymbol{\Theta}') \tag{7}$$

Since a peak intensity pair is either cooperative or non-cooperative, we can write $P(X_i = x_i, Y_i = y_i) = \pi_0 p_0(x_i, y_i; \boldsymbol{\theta}_0) + \pi_1 p_1(x_i, y_i; \boldsymbol{\theta}_1)$, where $\pi_0 + \pi_1 = 1$. Since we consider (X_i, Y_i) and (X_j, Y_j) $(i \neq j)$ to be statistically independent,

$$\log P(\mathbf{D} | \boldsymbol{\Theta}) = \sum_{i=1}^{N} \log \left(\pi_0 p_0^X(x_i; \boldsymbol{\theta}_0^X) p_0^Y(y_i; \boldsymbol{\theta}_0^Y) + \pi_1 p_1^X(x_i; \boldsymbol{\theta}_1^X) p_1^Y(y_i; \boldsymbol{\theta}_1^Y) \right)$$

$P(\mathbf{L}|\mathbf{D},\boldsymbol{\Theta})$ in equation (7) can be expanded as –

$$\begin{aligned} P(\mathbf{L}|\mathbf{D},\boldsymbol{\Theta}) &= \prod_{i=1}^{N} P(L_i = l_i|\mathbf{D},\boldsymbol{\Theta}) \\ &= \prod_{i=1}^{N} \frac{\pi_{l_i} p_{l_i}(x_i, y_i; \boldsymbol{\theta}_{l_i})}{\pi_0 p_0(x_i, y_i; \boldsymbol{\theta}_0) + \pi_1 p_1(x_i, y_i; \boldsymbol{\theta}_1)}, \end{aligned}$$

where l_i is 0 or 1. Substituting the above two expansions into the expression for Q in equation (7), it can be shown that Q simplifies to the form shown below, where it is a sum over only N terms (page 4 in [16])

$$\begin{aligned} Q(\boldsymbol{\Theta},\boldsymbol{\Theta}') &= \sum_{i=1}^{N}\sum_{l=1}^{2} \log(\pi_l) P(L_i = l|x_i, y_i, \boldsymbol{\Theta}') + \sum_{i=1}^{N}\sum_{l=1}^{2} P\big(L_i = l|x_i, y_i, \boldsymbol{\Theta}\big) \log\Big(p_l^X(x_i; \boldsymbol{\theta}_l^{X'}) p_l^Y(y_i; \boldsymbol{\theta}_l^{Y'})\Big) \\ &= Q_1(\boldsymbol{\Theta}') + Q_2(\boldsymbol{\Theta},\boldsymbol{\Theta}'), \end{aligned} \tag{8}$$

where, $\boldsymbol{\Theta}' = (\pi_0', \boldsymbol{\theta}_0^{X'}, \boldsymbol{\theta}_0^{Y'}, \boldsymbol{\theta}_1^{X'}, \boldsymbol{\theta}_1^{Y'})$. Note that the first term is independent of $\boldsymbol{\Theta}'$, so it can be maximized independently of the second term. The choice of π_l that maximizes the first term (page 5 in [16]) is –

$$\pi_l = \frac{1}{N}\sum_{i=1}^{N} P(L_i = l|x_i, y_i, \boldsymbol{\Theta}') \text{ for } l = 0, 1.$$

The $k-th$ EM iteration involves choosing a value $\boldsymbol{\Theta} = \boldsymbol{\Theta}^{(k+1)}$ that maximizes $Q(\boldsymbol{\Theta},\boldsymbol{\Theta}^{(k)})$, where $\boldsymbol{\Theta}^{(k)}$ is kept fixed at the value obtained in the previous EM iteration that maximizes $Q(\boldsymbol{\Theta},\boldsymbol{\Theta}^{(k-1)})$. EM involves two steps, an E-step and an M-step, which are both needed to maximize $Q(\boldsymbol{\Theta},\boldsymbol{\Theta}^{(k)})$. The E- and M- steps evaluated at the $i-th$ iteration in our algorithm are [17] –

- E-step : Compute

$$P(L_i = l|x_i, y_i, \boldsymbol{\Theta}^{(k)}) = \frac{{\pi_l}^{(k)} p_l(x_i, y_i; \boldsymbol{\Theta}^{(k)})}{{\pi_0}^{(k)} p_0(x_i, y_i; \boldsymbol{\Theta}^{(k)}) + {\pi_1}^{(k)} p_1(x_i, y_i; \boldsymbol{\Theta}^{(k)})} \text{ for } l = 0, 1;\ i = 1, 2, \ldots, N.$$

- M-step (1) : Compute

$$\pi_l^{(k+1)} = \sum_{i=1}^{N} P(L_i = l|x_i, y_i, \boldsymbol{\Theta}^{(k)}) \text{ for } l = 0, 1$$

  This step maximizes Q_1 in equation (8).

- M-step (2) : Use Powell's gradient search method, as implemented in the Scipy optimization toolbox [18] to find $\boldsymbol{\Theta}^{(k+1)}$ that maximizes $Q_2(\boldsymbol{\Theta},\boldsymbol{\Theta}^{(k)})$ in equation (8), with $\boldsymbol{\Theta}^{(k)} = (\pi_0^{(k)}, \boldsymbol{\theta}_0^{X,(k)}, \boldsymbol{\theta}_0^{Y,(k)}, \boldsymbol{\theta}_1^{X,(k)}, \boldsymbol{\theta}_1^{Y,(k)})$ kept constant –

$$Q_2(\boldsymbol{\Theta},\boldsymbol{\Theta}^{(k)}) = \sum_{i=1}^{N}\sum_{l=1}^{2} P\big(L_i = l|x_i, y_i, \boldsymbol{\Theta}\big) \log\Big(p_l^X(x_i; \boldsymbol{\theta}_l^{X,(k)}) p_l^Y(y_i; \boldsymbol{\theta}_l^{Y,(k)})\Big)$$

We terminate the EM algorithm after n iterations if

$$\frac{|Q(\boldsymbol{\Theta}^{(n)},\boldsymbol{\Theta}^{(n-1)}) - Q(\boldsymbol{\Theta}^{(n-1)},\boldsymbol{\Theta}^{(n-2)})|}{|Q(\boldsymbol{\Theta}^{(n-1)},\boldsymbol{\Theta}^{(n-2)})|} < 10^{-6}.$$

We choose the initial value $\boldsymbol{\Theta}^{(0)}$ as follows. From the data $\{(x_i, y_i)\}_{i=1}^N$, we separate the peak intensities of X and Y as $D_X = \{x_i\}_{i=1}^N$ and $D_Y = \{y_i\}_{i=1}^N$. We then compute the value $\boldsymbol{\theta}_{mle}^X$ that maximizes the likelihood $\prod_{i=1}^N p(x_i; \boldsymbol{\theta})$, where f is a Lognormal, Gamma or Gaussian density function. Similarly, we also compute the value of $\boldsymbol{\theta}_{mle}^Y$ that maximizes the likelihood $\prod_{i=1}^N p(y_i; \boldsymbol{\theta})$. These maximum likelihood estimates $\boldsymbol{\theta}_{mle}^X$ and $\boldsymbol{\theta}_{mle}^Y$ are computed using the `fit` function

provided by the Python Scipy `stats` library, which can provide maximum likelihood estimates when p_0 and p_1 are either Lognormal, Gamma or Gaussian density functions. We choose $\pi_0^{(0)}$ from a Uniform[0, 1] distribution. We finally set our initial parameter vector $\boldsymbol{\Theta}^{(0)}$ to $(\pi_0^{(0)}, \theta_{mle}^X, \theta_{mle}^Y, \theta_{mle}^X, \theta_{mle}^Y)$. We verified that EM converged to the same local maximum when $\boldsymbol{\Theta}^{(0)}$ was perturbed by up to 30% around this choice (data not shown).

I Calculation of receiver operating characteristic (ROC) curves

CPI-EM : Given probability of cooperative binding $p_1^{coop}, p_2^{coop}, \ldots, p_N^{coop}$, the ROC curve was calculated by picking thresholds α on these probabilities that corresponded to false positive rates between 0.1 and 1 in steps of 0.1. The true positive rate at each of these thresholds was then computed. The area under the ROC curve was then calculated using the trapezoidal integration rule available in the Python `numpy` library. This procedure was repeated for each of the three variants of the CPI-EM algorithm. Similarly, the ROC curve of the peak distance algorithm was computed by choosing thresholds on the peak distance that corresponded to false positive rates between 0.1 and 1 in steps of 0.1. After the true positive rate at each threshold was calculated, the area under the ROC was computed with the trapezoidal integration rule.

STAP : Given the STAP cooperative indices at each location in the test dataset $\Delta_1, \Delta_2, \ldots, \Delta_n$ (see Materials and Methods in main text for how these indices are calculated), we declare those locations where Δ exceeds a threshold T as cooperatively bound. To compute the ROC, we choose values of T that correspond to false positive rates between 0.1 and 1 in steps of 0.1, and compute the true positive rate at each of these values. The area under the ROC is finally computed using the trapezoidal integration rule in a manner identical to that employed for computing the area under ROC of CPI-EM.

J Area under ROC of a chance detector is 0.5

The chance detector is based purely on using tosses from a biased coin to detect cooperative interactions. Let the probability of the coin showing heads be α. Out of a set of N peak intensity pairs $\{(x_i, y_i)\}$, suppose there are N_c and N_{nc} cooperatively and non-cooperatively bound pairs, respectively. The number of false positives, resulting from N tosses of the coin, would be $N_{nc}\alpha$, while the number of true positives would be $N_c\alpha$. This means that both the FPR and TPR of the chance detector would be α. Thus, as α is varied between 0 and 1, the ROC of the chance detector will be the straight line $FPR = TPR = \alpha$, which encloses an area of 0.5.

We checked if the performance of CPI-EM was dependent on instances of indirect binding between target and partner TF peaks and the size of the ChIP-seq peaks input to it. We found that removing indirectly bound peaks of target and partner TFs (QA Fig) did not noticeably alter the auROCs of any of the CPI-EM variants, with the log-normal CPI-EM variant continuing to perform better than chance across all datasets. When we trimmed the peaks of both TFs to within 50 base pair on either side of the peak summits before passing them as inputs to CPI-EM, there was no noticeable drop in the auROCs of any of the CPI-EM variants, except in the case of the mid-exponential phase CRP-FIS dataset. The auROC of the log-normal CPI-EM variant (QB Fig) on this dataset dropped below 0.5.

K Calculation of precision-recall curves

Precision-recall curves provide an alternate method to determining the performance of a detection algorithm. Using the terminology introduced in the main text, the precision (P) and recall R of an algorithm is

$$P = \frac{N_{TP}}{N_{TP} + N_{FP}},$$
$$R = \frac{N_{TP}}{N_{TP} + N_{FN}},$$

where, N_{TP} is the number of true positives, N_{FP} is the number of false positives and N_{FN} is the number of false negatives (i.e. the number of cooperatively bound peaks that are erroneously declared as non-cooperatively bound). As stated in the main text, these quantities are dependent on the detection threshold employed in the algorithm. Changing the threshold over a range gives a series of precision and recall values, with the area under the precision-recall curve (referred to as the average precision), providing a measure of detection performance.

We computed the average precision and precision-recall curves of both CPI-EM and STAP (N Fig) using the `average_precision_score` and `precision_recall_curve` routines from the `scikit-learn` library (v0.19.1) [27]. For the precision-recall curve of CPI-EM, we passed the probabilities of cooperative binding $p_1^{coop}, p_2^{coop}, \ldots, p_N^{coop}$ as inputs to these two routines. In the case of STAP, we passed the cooperative indices $\Delta_1, \Delta_2, \ldots, \Delta_n$ to compute its precision-recall curve.

L Estimation of mutual information

Given a probability distribution $p(x, y)$ over the set of peak intensity pairs $\{(x_i, y_i)\}_{i=1}^N$, mutual information (MI) is calculated as

$$MI = \sum_{i=1}^{n} \sum_{i=1}^{n} p(x_i, y_i) \log_2 \left(\frac{p(x_i, y_i)}{p_X(x_i) p_Y(y_i)} \right),$$

where $p_X(y)$ and $p_Y(y)$ are the marginal distributions of $p(x, y)$, and n is the number of $\{(x_i, y_i)\}$ pairs. MI is a non-negative quantity whose value is zero if X and Y are statistically independent i.e. if $p(x, y) = p_X(x)p_Y(y)$. From the knockout data available for each of the TF pairs, we separated the peak intensity pairs $\{(x_i, y_i\}_{i=1}^N$ into a set of cooperatively bound peak intensity pairs $A_c = \{(x_j, y_j)\}$ and a set of non-cooperatively bound peak intensity pairs $A_{nc} = \{(x_k, y_k)\}$. We separately computed the MI of peak intensity pairs in A_c (setting $p = p_1$ in equation (L)) and A_{nc} (setting $p = p_0$ in equation (L)) in each ChIP-seq knockout dataset we analyzed (Table G).

Estimating mutual information (MI) through direct use of the definition specified by equation (L) leads to many problems; such MI estimates can be biased, or go to infinity in the case of certain distributions [13]. We estimated MI using the LNC algorithm implemented in [13] that circumvents these issues. The drawback of the LNC algorithm was that it gave non-negative estimates of MI only when a sufficient number of data points (in our case, at least 20 peak pairs) were present and if the true value of MI is not too low [13] . We were thus unable to reliably estimate MI in some of the ChIP-seq datasets we analyzed. The code for the LNC algorithm is available at https://github.com/BiuBiuBiLL/NPEET_LNC/blob/master/lnc.py

Our finding that the mutual information between target and partner TF peak intensities is low, irrespective of whether they are cooperatively or non-cooperatively bound (Table G) reflects only on the low statistical dependence between both distribution of peak intensities. Importantly, this does not necessarily contradict our other finding that target TF peaks are more weakly bound when that are cooperatively bound, in comparison to regions where they are not cooperatively bound. The example below demonstrates that both these findings can be consistent with each other —

Suppose A and B are target and partner TFs, and the intensities of A and B are either 1 or 2, with "1" representing weak binding and "2" representing strong binding. Our statement that target TFs are more weakly bound when they

Case I : When B cooperatively binds DNA with A.

	B = 1	B = 2	
A = 1	0.18	0.42	P(A = 1) = 0.6
A = 2	0.12	0.28	P(A = 2) = 0.4
	P(B = 1) = 0.3	P(B = 2) = 0.7	

Case II : When B does not cooperatively bind DNA with A.

	B = 1	B = 2	
A = 1	0.1	0.1	P(A = 1) = 0.2
A = 2	0.4	0.4	P(A = 2) = 0.8
	P(B = 1) = 0.5	P(B = 2) = 0.5	

Table I: Probabilities of observing peak intensities of A and B in regions where (I) B cooperatively binds DNA with A, and (II) B does not cooperatively bind DNA with A

are cooperatively bound with B, when compared to regions where they are not cooperatively bound can be written as

$$P(\text{A} = 1|\text{B cooperatively binds DNA with A}) > P(\text{A} = 1|\text{B does not cooperatively binds DNA with A}).$$

Two sets of probabilities that satisfy this statement are shown in Table I, where each cell represents the probability of observing a given peak intensity for A and B. For example, in sub-table I of Table I, the entry in the first row, first column states that $P(\text{A} = 1 \text{ and B} = 1|\text{B cooperatively binds DNA}) = 0.18$. From the margins of both tables, we see that

$$P(A = 1) = 0.6, P(A = 2) = 0.4 \text{ (when B cooperatively binds DNA with A)}$$
$$P(A = 1) = 0.2, P(A = 2) = 0.8 \text{ (when B does not cooperatively bind DNA with A)}$$

and

$$P(B = 1) = 0.3, P(B = 2) = 0.7 \text{ (when B cooperatively binds DNA with A)}$$
$$P(B = 1) = 0.5, P(B = 2) = 0.5 \text{ (when B does not cooperatively bind DNA with A)}$$

Thus, the statement that A is more likely to be weakly bound when it is cooperatively bound with B is satisfied.

The statement about mutual information being low shows that the peak intensities of A and B are statistically independent. In our example, the mutual information between peak intensities of A and B is computed as

$$\begin{aligned} MI = {} & P(A = 1 \text{ and } B = 1|E) \log_2 \left(\frac{P(A = 1 \text{ and } B = 1|E)}{P(A = 1|E) \times P(B = 1|E)} \right) \\ & + P(A = 1 \text{ and } B = 2|E) \log_2 \left(\frac{P(A = 1 \text{ and } B = 2|E)}{P(A = 1|E) \times P(B = 2|E)} \right) \\ & + P(A = 2 \text{ and } B = 1|E) \log_2 \left(\frac{P(A = 2 \text{ and } B = 1|E)}{P(A = 2|E) \times P(B = 1|E)} \right) \\ & + P(A = 2 \text{ and } B = 2|E) \log_2 \left(\frac{P(A = 2 \text{ and } B = 2|E)}{P(A = 2|E) \times P(B = 2|E)} \right) , \end{aligned}$$

where E refers to the distribution corresponding to the values in sub-tables I or II in Table I. It can be seen that the MI will be zero if the ratios within the logarithms are equal to 1, that is —

$$P(A = i \text{ and } B = j \,|\, E) = P(A = i \,|\, E) \times P(B = j \,|\, E), \tag{9}$$

where i and j are either 1 or 2. The values chosen in Table I are such that the equalities in equation (9) will always hold.

We demonstrate this below for the case when $A = 2$ and $B = 1$, and both A and B cooperatively bind DNA.

$$\begin{aligned} P(\text{A} = 2 \,|\, \text{B cooperatively binds with A}) \times P(B = 1 \,|\, \text{B cooperatively binds with A}) \\ &= 0.8 \times 0.5 \\ &= 0.4 \\ &= P(\text{A} = 2 \text{ and B} = 1 | \text{B cooperatively binds with A}). \end{aligned}$$

Thus, in this example, we can see that the MI between the peak intensities of A and B is zero, and at the same time, A is more weakly bound when it is cooperatively bound with B when compared to regions where A is not cooperatively bound.

In more intuitive terms, that a target TF tended to be more weakly bound when it cooperatively bound DNA, means that we are more likely to find the target TF peak intensity to be low if we are told that it is cooperatively bound by the partner TF. While it is true that a target TF should have a higher affinity to DNA when cooperatively binding with a partner TF, this implicitly assumes that binding site affinities of the target TF are, on average, the same between cooperatively and non-cooperatively bound regions. However, in F Fig, we see that the motif scores of target TF peaks are typically lower in regions where the target TF is cooperatively bound. Thus, it appears that target TF binding affinities are intrinsically lower in cooperatively bound regions than in non-cooperatively bound regions. Though in G Fig, we see that the target TF motif scores and target TF peak intensities are not always correlated (in terms of their Pearson correlation coefficient), this means that this is not the sole reason as to why peak intensities of the target TF are low in cooperatively bound regions.

The low value of MI reflects the statistical independence between the peak intensities of TFs that cooperatively bind DNA. The consequence of this is that the probability of finding a target TF to be weakly bound is independent of the peak intensity of the partner TF, and that this is true whether or not the two are cooperatively bound. Put differently, if we were asked to guess if the target TFs peak intensity at a particular location was low or high, then, the accuracy of our guess will remain the same even if we were told the peak intensity of the partner TF. Thus, the mutual information refers to the probability of finding a target TF to be weakly bound based on knowledge of the partner TFs peak intensity, and does not refer to the actual peak intensity of the target TF.

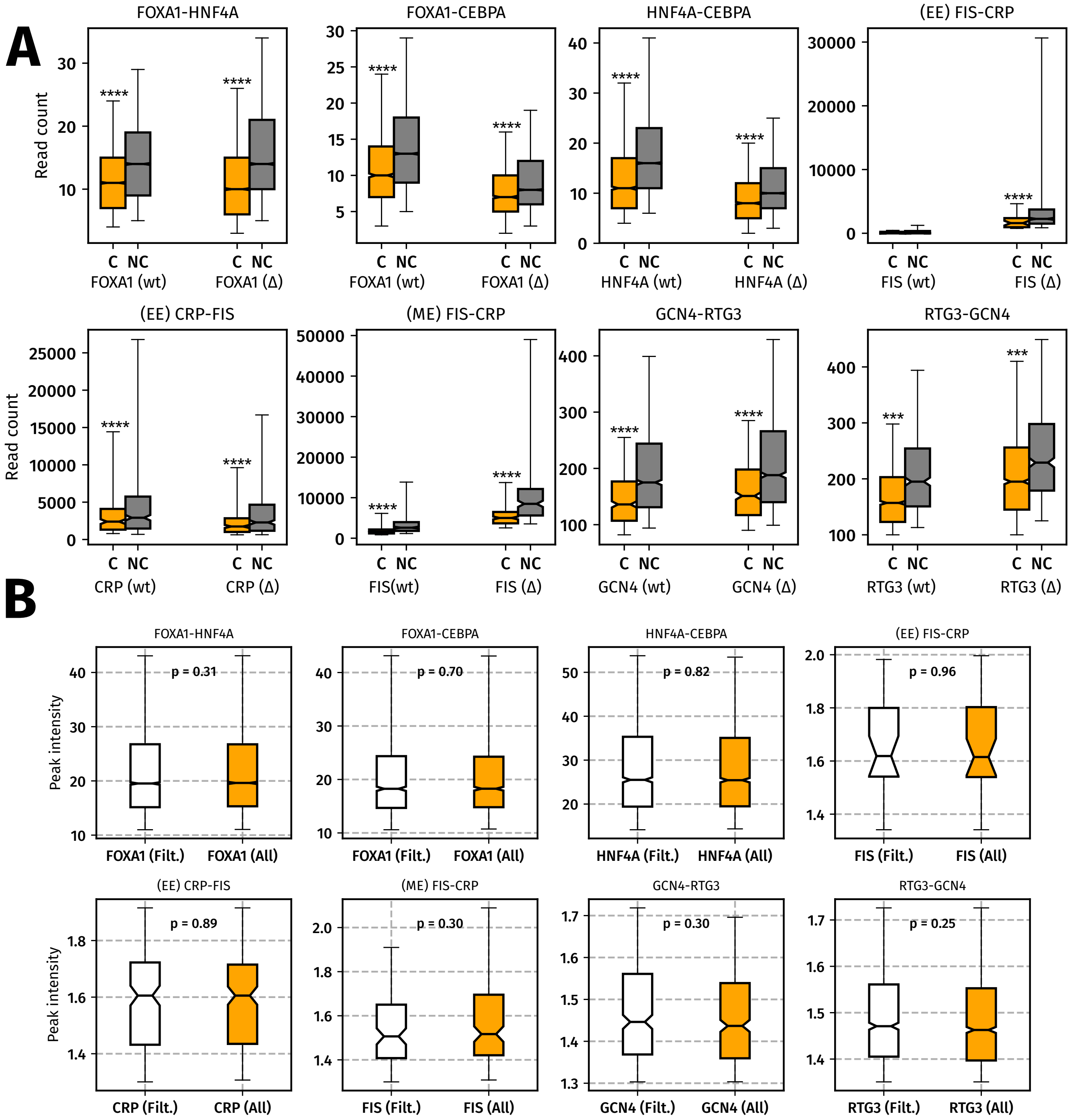

Figure A: (A) The read count in ChIP-seq input from cooperatively bound regions is significantly lower than in non-cooperatively bound regions. Each panel contains a pair of boxplots of the number of reads falling within cooperatively bound (C, in orange) and non-cooperatively bound (NC, in black) peak region of the target TF from the ChIP-seq input of wild-type (wt) cells and cells where the indicated partner TF is knocked out (Δ). The whiskers represent the 5th and 95th percentiles of the distribution. **** and *** indicate p-values of less than 10^{-4} and 10^{-3}, respectively, from a Wilcoxon rank-sum test. **(B) However, this difference in input read count distributions does not affect the intensity distribution of cooperatively bound peaks.** In each panel, the white boxplot is the intensity distribution of those cooperatively bound target TF peaks whose input read counts fall within the 5th and 95th percentile of the input read count distribution of non-cooperatively bound target TF peaks. The orange boxplot is the intensity distribution of all cooperatively bound peaks, which is identical to the boxplots of the target TF shown in orange in Figure in the main text. The p-values in each panel are calculated from a Wilcoxon rank sum test between the white and orange distributions in each panel.

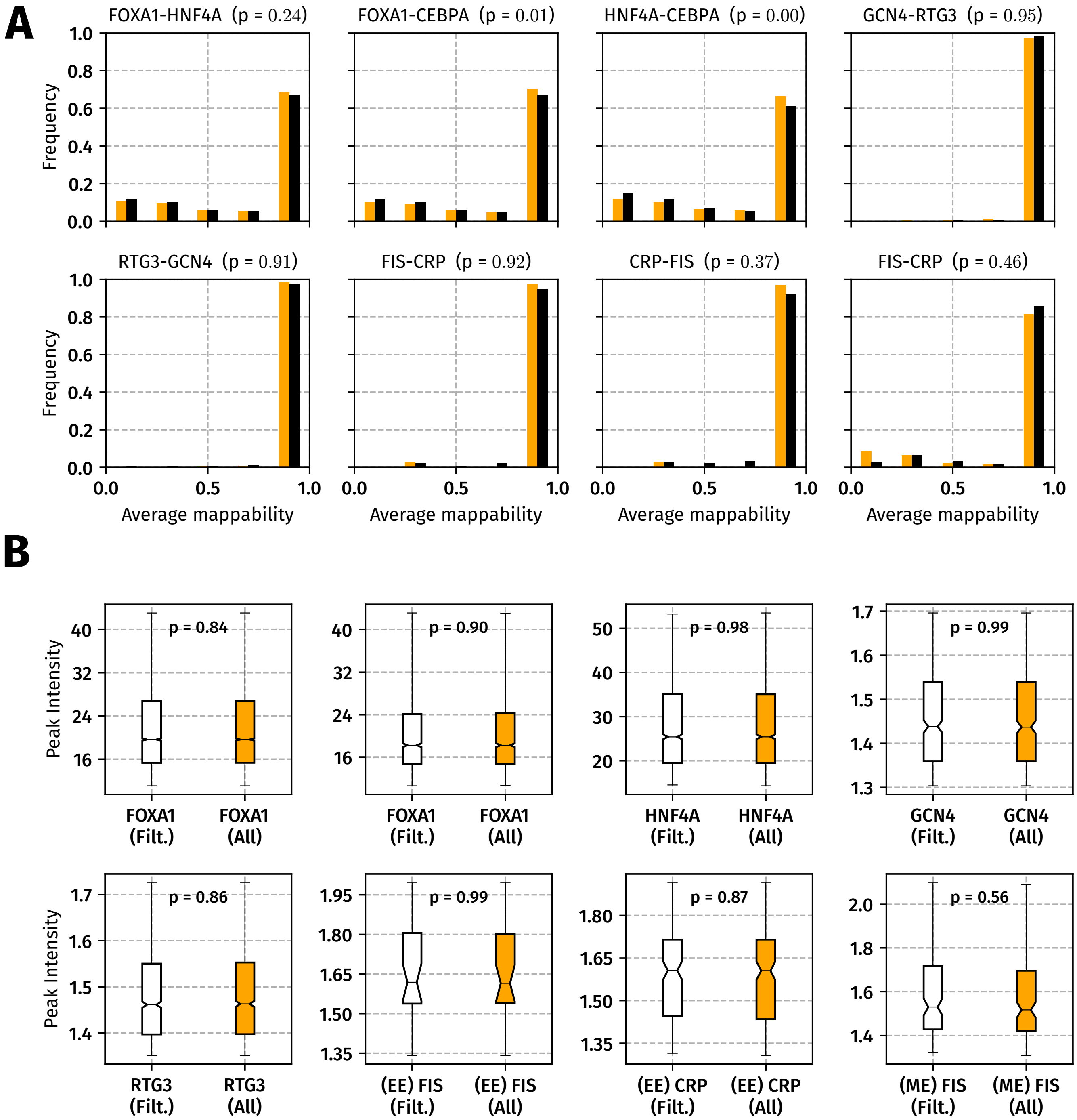

Figure B: (A) The average mappability of cooperatively bound regions differs from those of non-cooperatively bound regions in some data sets. Each panel contains a pair of histograms of the average mappability of cooperatively bound (orange) and non-cooperatively bound (black) peaks of the target TF. The p-values between the two histograms in each plot are from a Wilcoxon rank-sum test. Aside from the FOXA1-CEBPA and HNF4A-CEBPA datasets, the average mappability between cooperatively and non-cooperatively bound target TF peaks are not significantly different. See C Section for the calculation of average mappability. **(B) Differences in average mappability do not affect the intensity distribution of cooperatively bound peaks**. In each panel, the white boxplot is the intensity distribution of those cooperatively bound target TF peaks whose average mappability falls within the 5th and 95th percentile of the average mappability distribution of non-cooperatively bound target TF peaks. The orange boxplot is the intensity distribution of all cooperatively bound peaks, which is identical to the boxplots of the target TF shown in orange in Figure 1 in the main text. The p-values in each panel are calculated from a Wilcoxon rank sum test between the white and orange distributions in each panel.

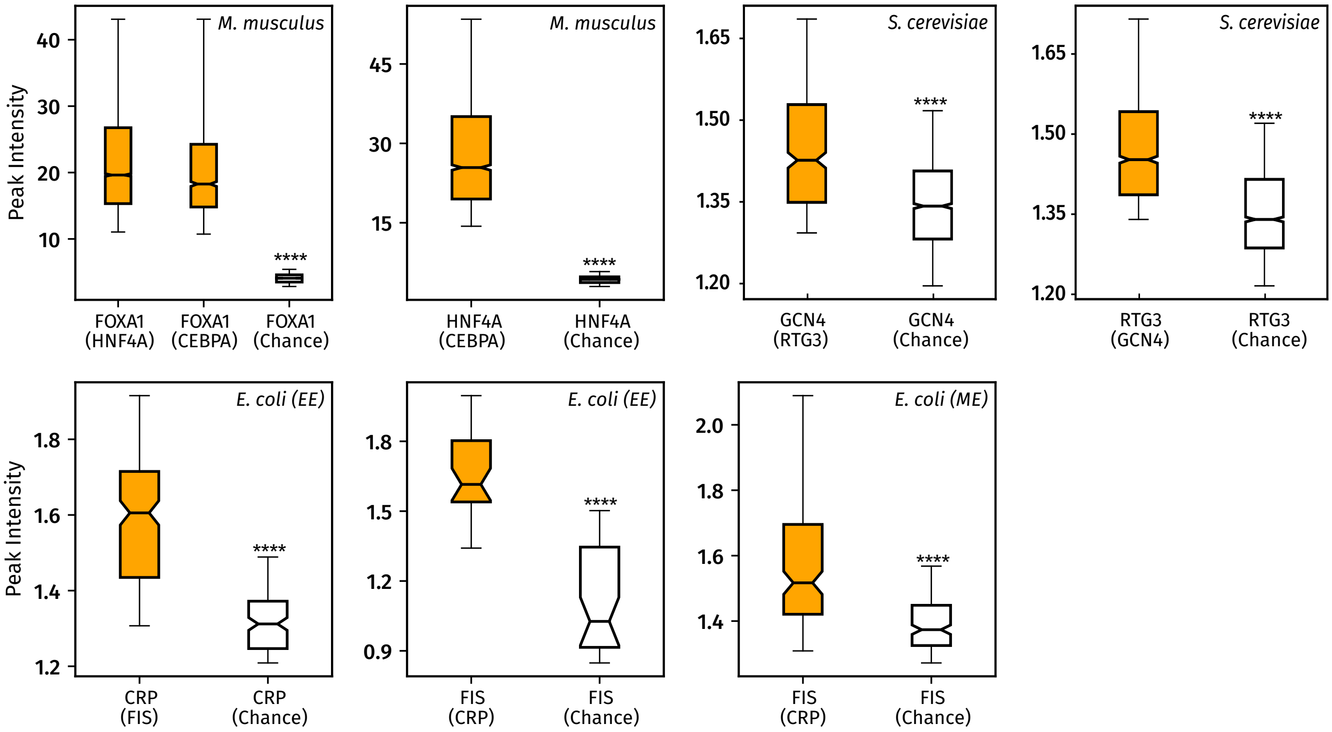

Figure C: Intensities of cooperatively bound peaks are significantly higher than the intensities of peaks that are lost between replicates In each panel, the boxplots in orange are intensities of cooperatively bound target TFs, which are identical to the boxplots of cooperatively bound target TFs shown in Figure 1 in the main text. The boxplots in white are intensities of those target TF peaks that are present in the merged alignments of all replicates but are absent in each of the individual replicates. **** indicates p-values of less than 10^{-4} as computed by the Wilcoxon rank sum test. The whiskers of each box plot represents the 5th and 95th percentile of that distribution.

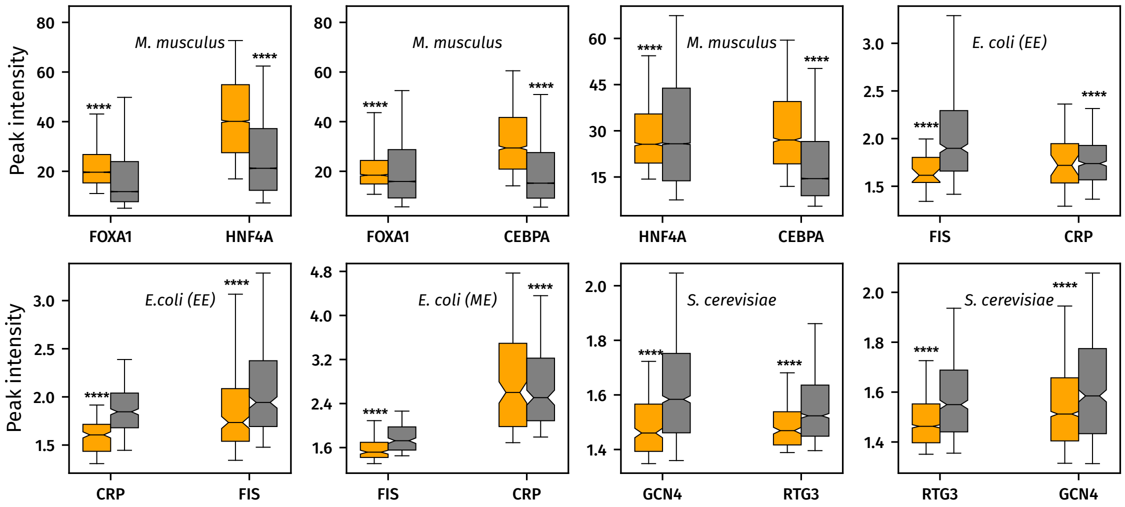

Figure D: Peak intensities of cooperatively bound target TFs are significantly lower than those of non-cooperatively bound target TFs after peaks have been trimmed to 50 base pairs on either side of their summit. Box-plots of peak intensity distributions of cooperatively (orange) and non-cooperatively (gray) bound TF pairs, with target TFs on the left and partner TFs on the right. **** and *** indicates p-values of $< 10^{-4}$ and $< 10^{-3}$, respectively, from the Wilcoxon rank sum test. The whiskers of the box plot are the $5-th$ and $95-th$ percentiles of the distributions shown.

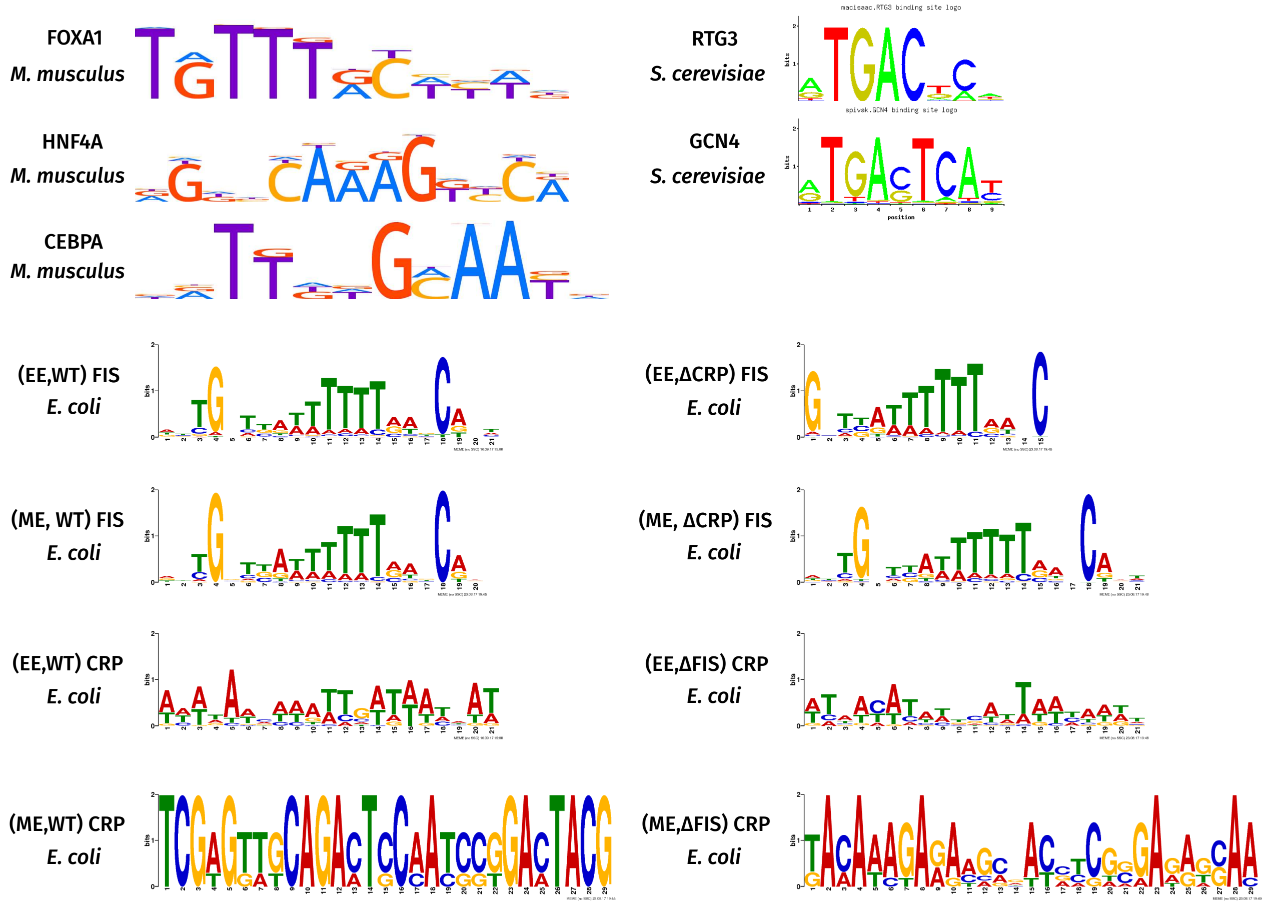

Figure E: Motifs used in all sequence-based analyses of ChIP-seq data in Figure 2 of the main text. The motifs of FOXA1, HNF4A and CEBPA in *M. musculus* ChIP-seq data were sourced from the HOCOMOCO v10 database [19]. The motifs of GCN4 and RTG3 were sourced from the ScerTF database [20]. The motifs of CRP and FIS were learnt de novo using the MEME suite (see Materials and Methods in the main text).

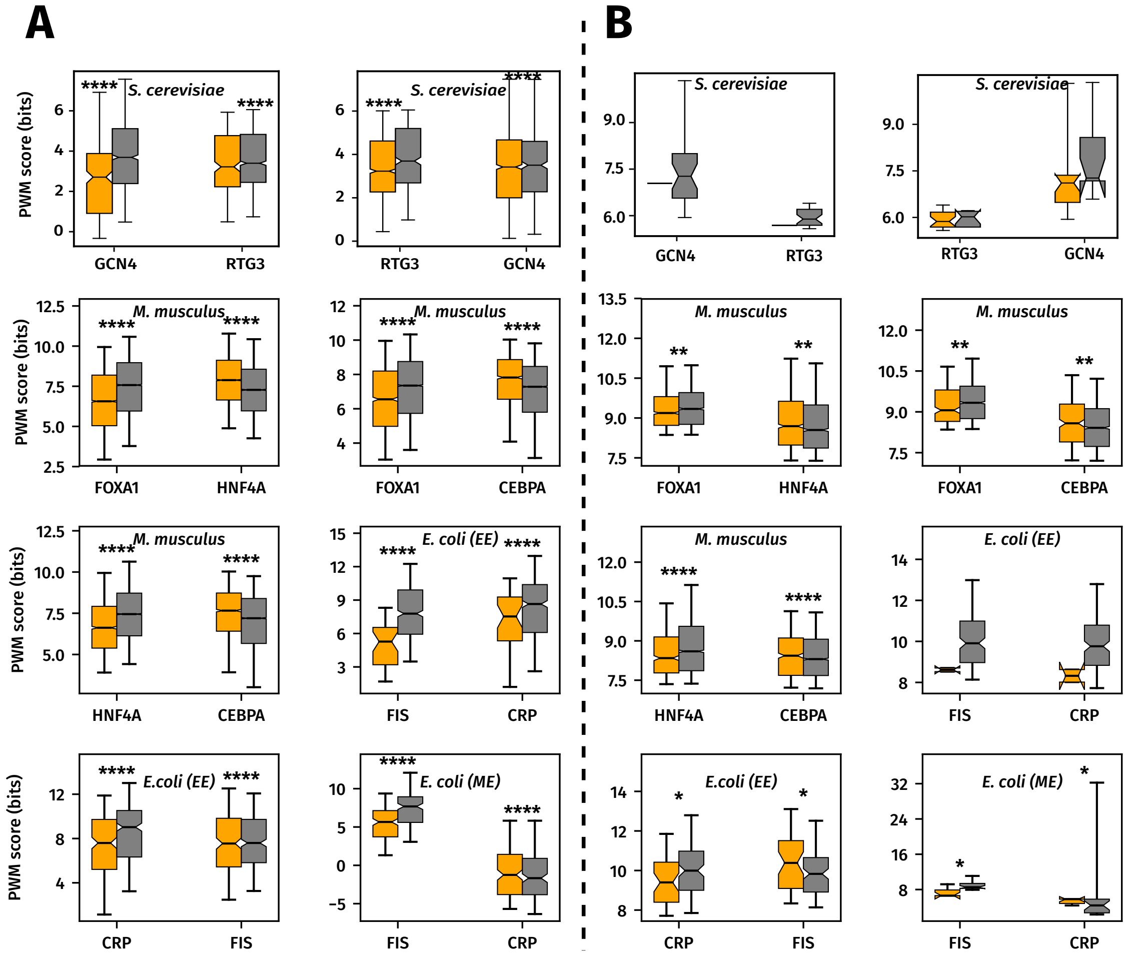

Figure F: Motif scores of cooperatively bound target TFs and non-cooperatively bound target TFs. Box-plots of motif score distributions of cooperatively (orange) and non-cooperatively (gray) bound TF pairs, with target TFs on the left and partner TFs on the right. **(A) Motif scores of cooperatively bound target TFs are lower than those of non-cooperatively bound target TFs.** ****, *** and ** indicate p-values of $< 10^{-4}$,$< 10^{-3}$ and 10^{-2} from a Wilcoxon rank sum test. The whiskers of the box plot are the $5-th$ and $95-th$ percentiles of the distributions shown. **(B) After indirectly bound ChIP-seq peaks are removed, the motif scores of the remaining cooperatively bound target TFs are lower than those of the remaining non-cooperatively bound target TF peaks.** In the *S. cerevisiae* and *E. coli* datasets, motif scores in cooperatively bound target peaks are not significantly lower than those of non-cooperatively bound peaks. However, this is partly because a large number of peaks were filtered out as indirectly bound in these datasets (E and F Tables).

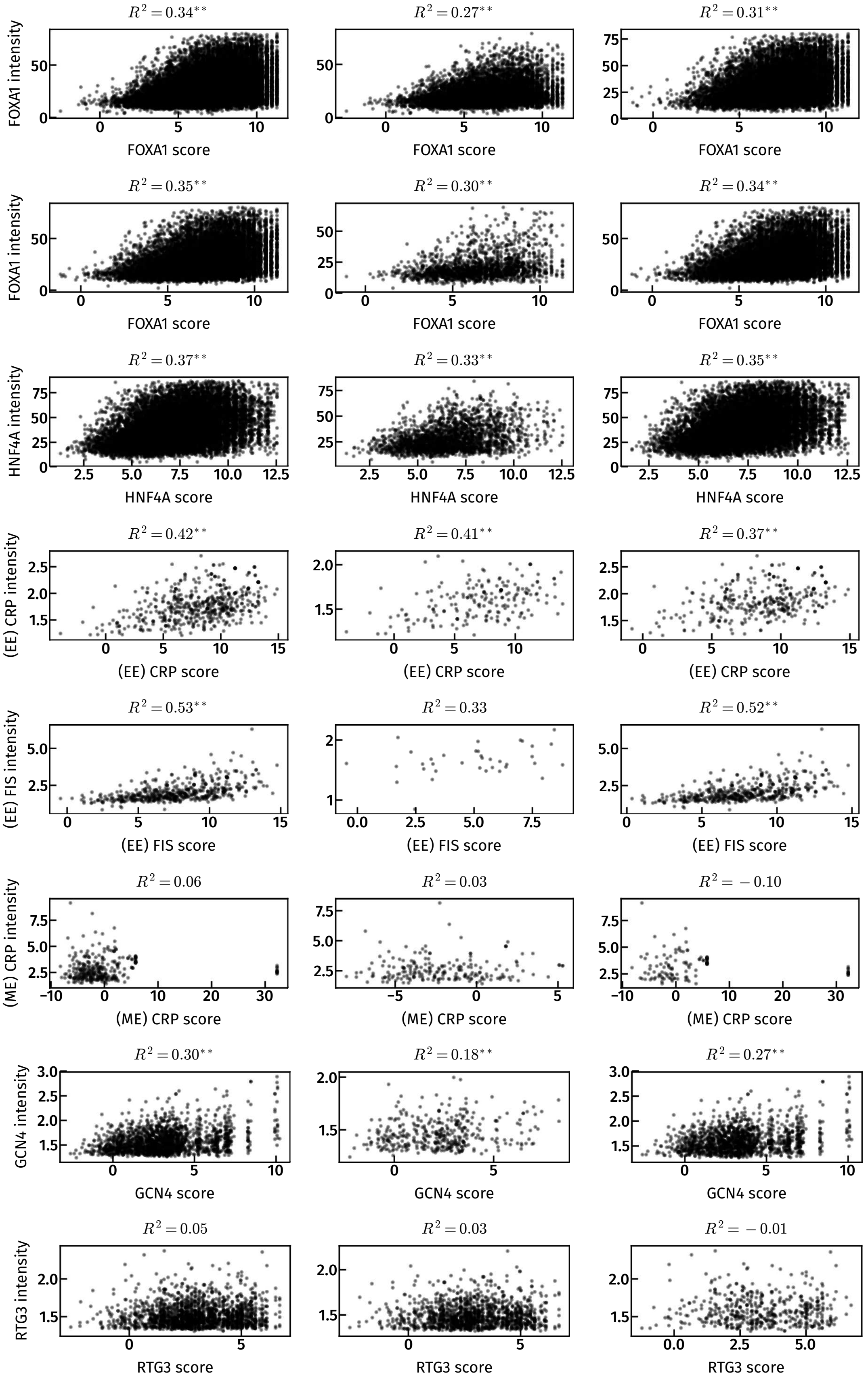

Figure G: Motif scores and peak intensities of the target TF are significantly correlated only in *M. musculus* datasets Each row represents a different dataset. Each panel is a scatter between the highest motif score in each peak region and the intensity of that peak, with the R^2 value representing the Pearson correlation coefficient between the motif scores and peak intensities of each dataset, with ** indicating a p-value of less than 0.01. The left column represents scatters of motif scores and peak intensities from all doubly bound regions across the genome, while the middle and the right columns are scatters from cooperatively bound regions and non-cooperatively bound regions, respectively. The R^2 value is statistically significant across all *M. musculus* datasets (FOXA1,HNF4A and CEBPA), but is true only in subsets of doubly bound locations in the remaining datasets.

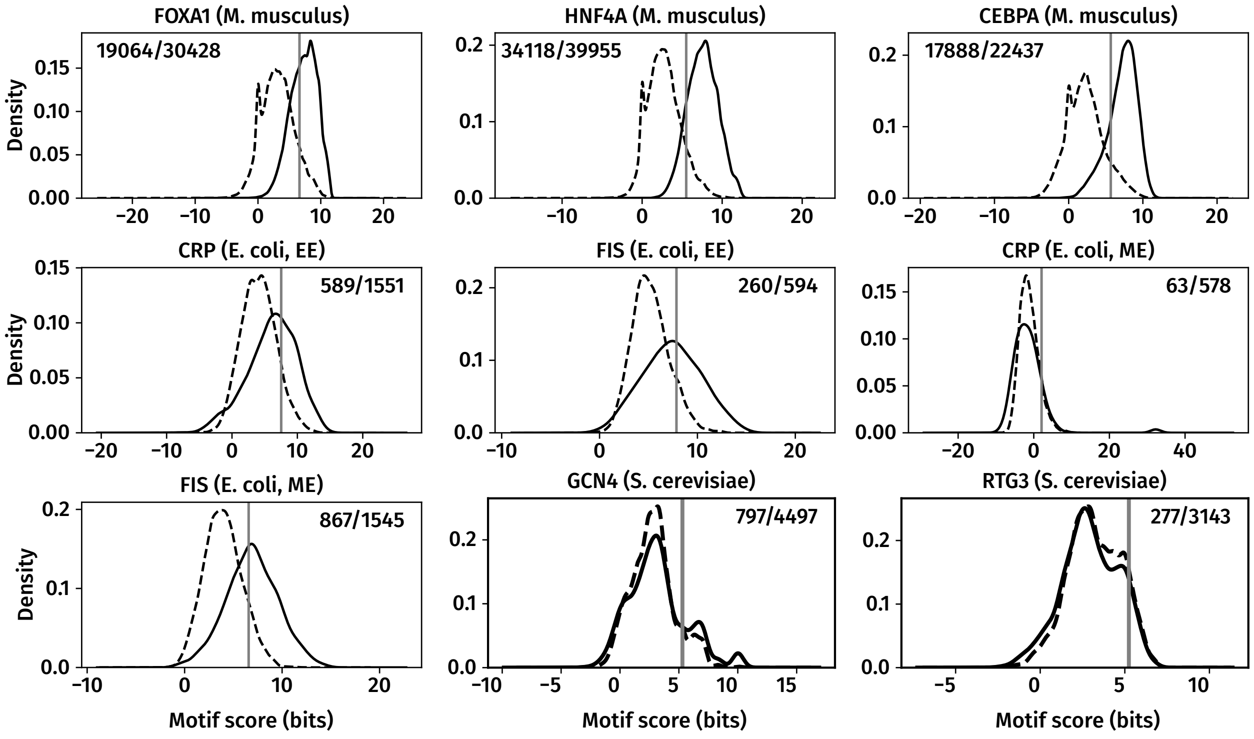

Figure H: Distributions of motif scores from sequences in ChIP-seq peaks (solid lines) and from unbound regions (dashed lines) used to detect indirectly bound peaks. The vertical gray line in each panel represents the $90-th$ percentile of the distribution of motif scores from unbound sequences. ChIP-seq peaks whose motif scores are less than the $90-th$ percentile are considered to be indirectly bound. The numbers in the top-right of each panel indicate the number of directly bound peaks and the total number of peak that clear the IDR/FDR criterion in each dataset.

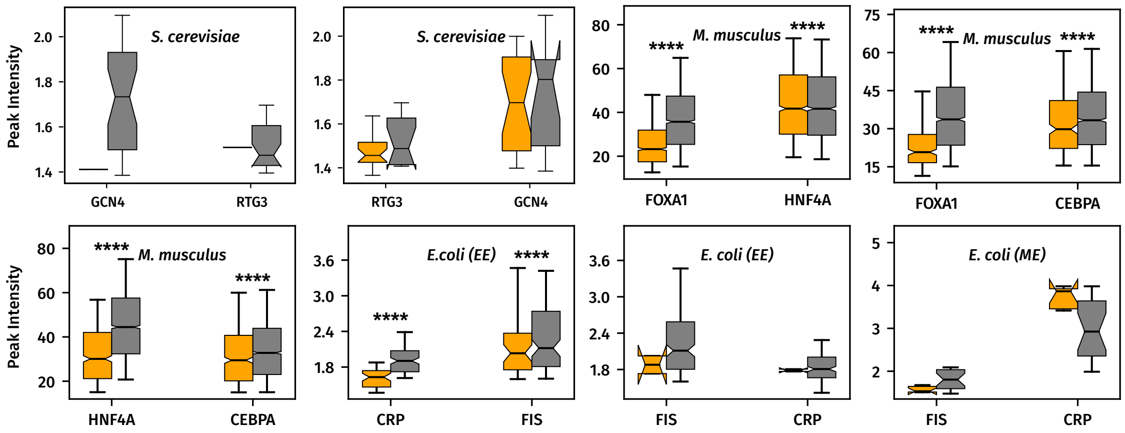

Figure I: After indirectly bound ChIP-seq peaks are removed, the peak intensities of the remaining cooperatively bound target TFs are lower than the peak intensities of the remaining non-cooperatively bound target TFs. Box-plots of peak intensity distributions of cooperatively (orange) and non-cooperatively (gray) bound TF pairs, with target TFs on the left and partner TFs on the right. **** indicates a p-value of $< 10^{-4}$ from a Wilcoxon rank sum test. The whiskers of the box plot are the $5-th$ and $95-th$ percentiles of the distributions shown. In *S. cerevisiae* and *E. coli* datasets, cooperatively bound target peak intensities are not significantly lower than those of non-cooperatively bound peaks. However, this is partly because a large number of peaks were filtered out as indirectly bound in these datasets (E and F Tables).

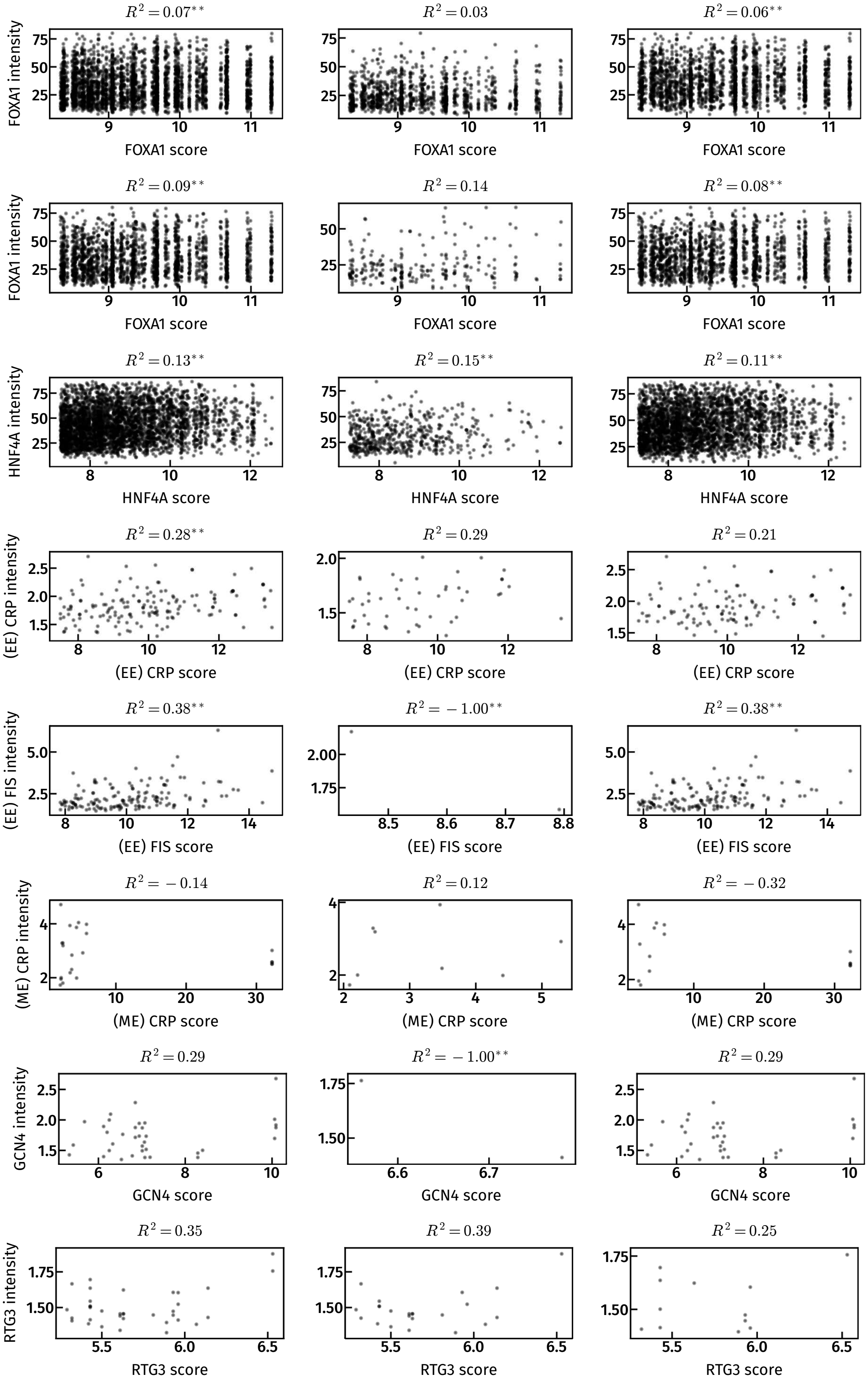

Figure J: After indirectly bound peaks are removed, motif scores and peak intensities of the target TF are not significantly correlated Each row represents a different dataset. Each panel is a scatter between the highest motif score in each peak region and the intensity of that peak, with the R^2 value representing the Pearson correlation coefficient between the motif scores and peak intensities of each dataset, with ** indicating a p-value of less than 0.01. The left column represents scatters of motif scores and peak intensities from all doubly bound regions across the genome, while the middle and the right columns are scatters from cooperatively bound regions and non-cooperatively bound regions, respectively.

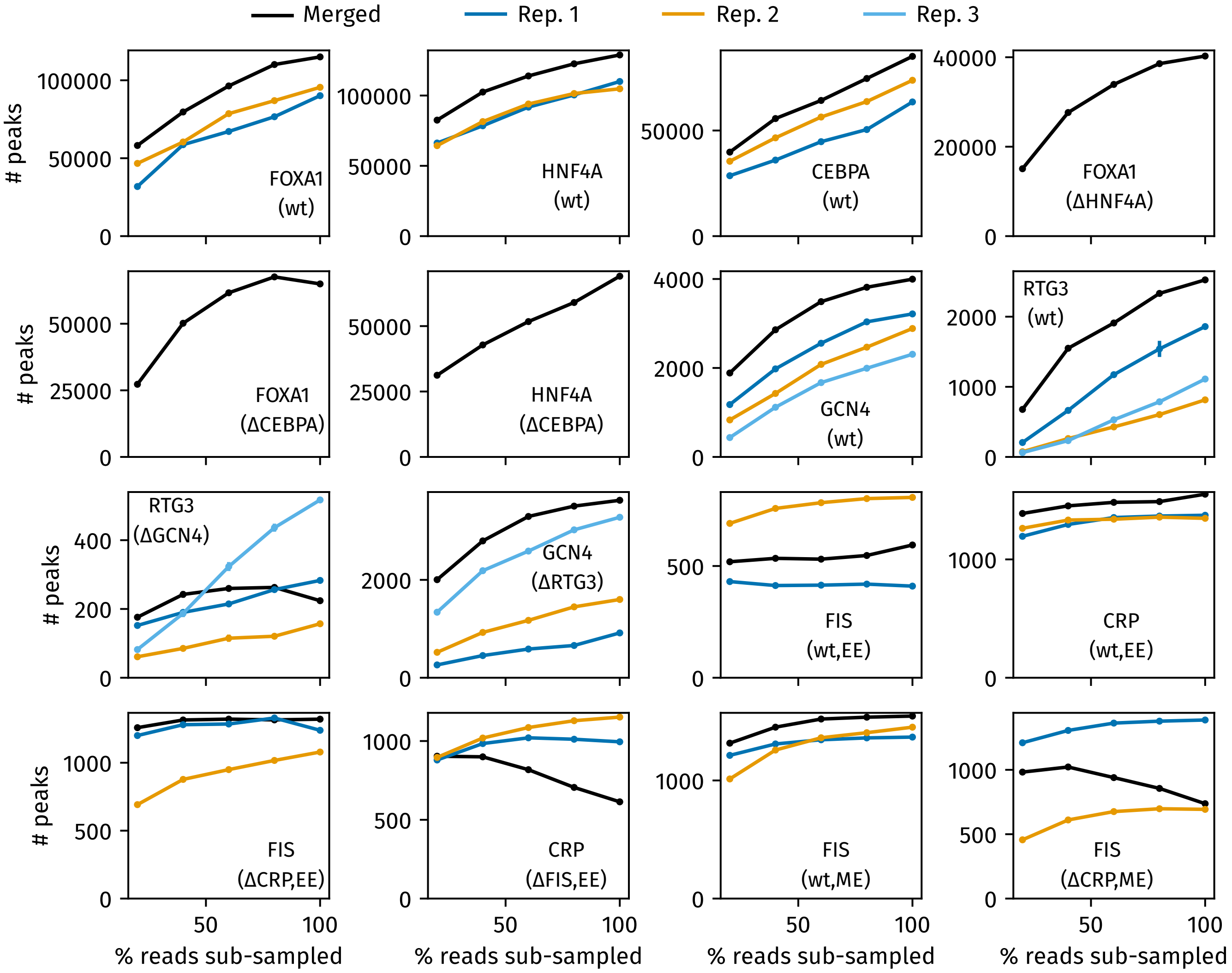

Figure K: An analysis of the dependence between the number of peaks called and the number of sequence reads in ChIP and input samples. For each ChIP-seq dataset, we sub-sampled between 20% and 80% of the aligned reads in ChIP and input samples (shown on the x-axis), and called peaks on each sub-sampled set (see F Section for more details) . This sub-sampling was done in three replicates for each fraction, and the error bars shown represent the standard deviation of the number of peaks at each sub-sampling fraction. The orange and blue lines represent individual replicate ChIP and input libraries, and the black lines represent peak calls from combining both replicates. There is only a single line for the FOXA1 (ΔHNF4A), FOXA1 (ΔCEBPA) and HNF4A (ΔCEBPA) datasets, since only a single replicate of sequencing was performed in the original experiments.

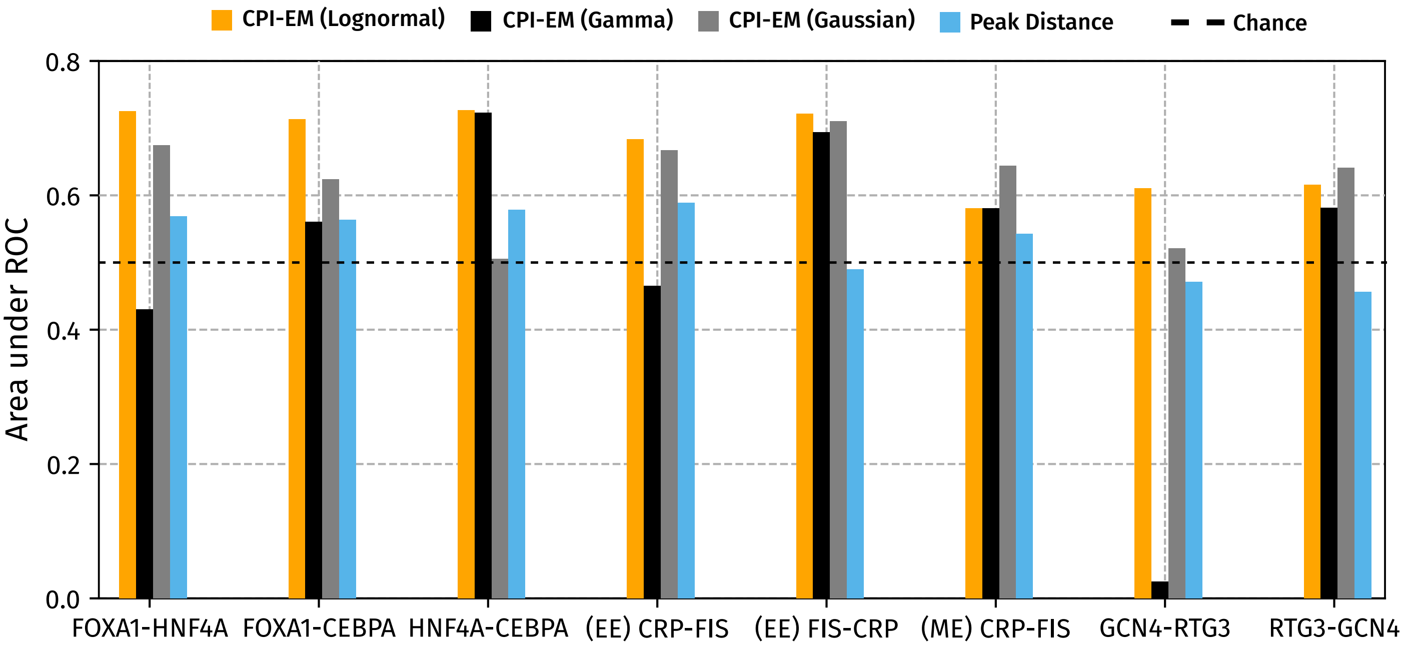

Figure L: The CPI-EM variant that fits Log-normal distributions to peak intensity pairs consistently performs well across all datasets The area under the ROC curve (auROC) of the CPI-EM algorithm applied to each of the datasets shown in Figure 1 in the main text. CPI-EM variants that fit Lognormal, Gamma and Gaussian distributions are represented in orange, black and gray, respectively. The auROC of the peak distance based detector is shown in blue, while the auROC of the chance detector is shown by a dashed line. See I Section for the calculation of the ROC curve for both the CPI-EM and peak distance algorithms. The complete ROC curves for these datasets are shown in Figure P

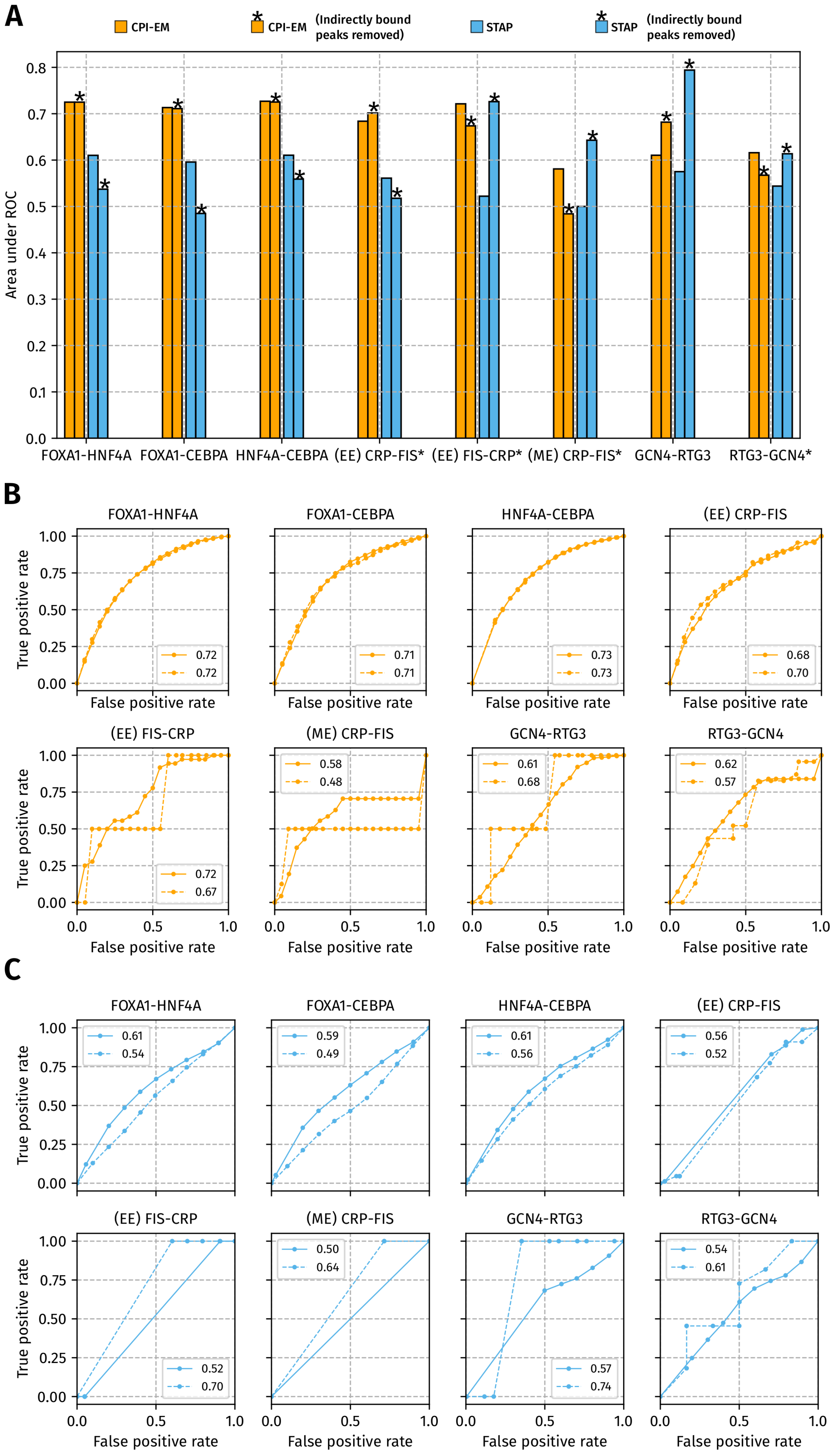

Figure M: There is only a minimal effect of removing indirectly bound peaks on the performance of both the Log-normal CPI-EM variant and STAP (A) The auROCs of STAP and CPI-EM are in sky blue and orange respectively, while the striped bars represent the auROCs after indirectly bound peaks are removed from the dataset. The datasets marked with an asterisk (*) are those where STAP was numerically unstable i.e. it did not consistently converge to the same model parameters after multiple runs. **(B,C)** The complete ROC curves of both STAP and CPI-EM for each of the datasets in (A). The ROC curves of CPI-EM and STAP are in orange and sky blue, respectively. The dashed lines indicate that indirectly bound peaks have been removed from the data before they have been input to both algorithms. The numbers in the legend indicate the ROC of each algorithm.

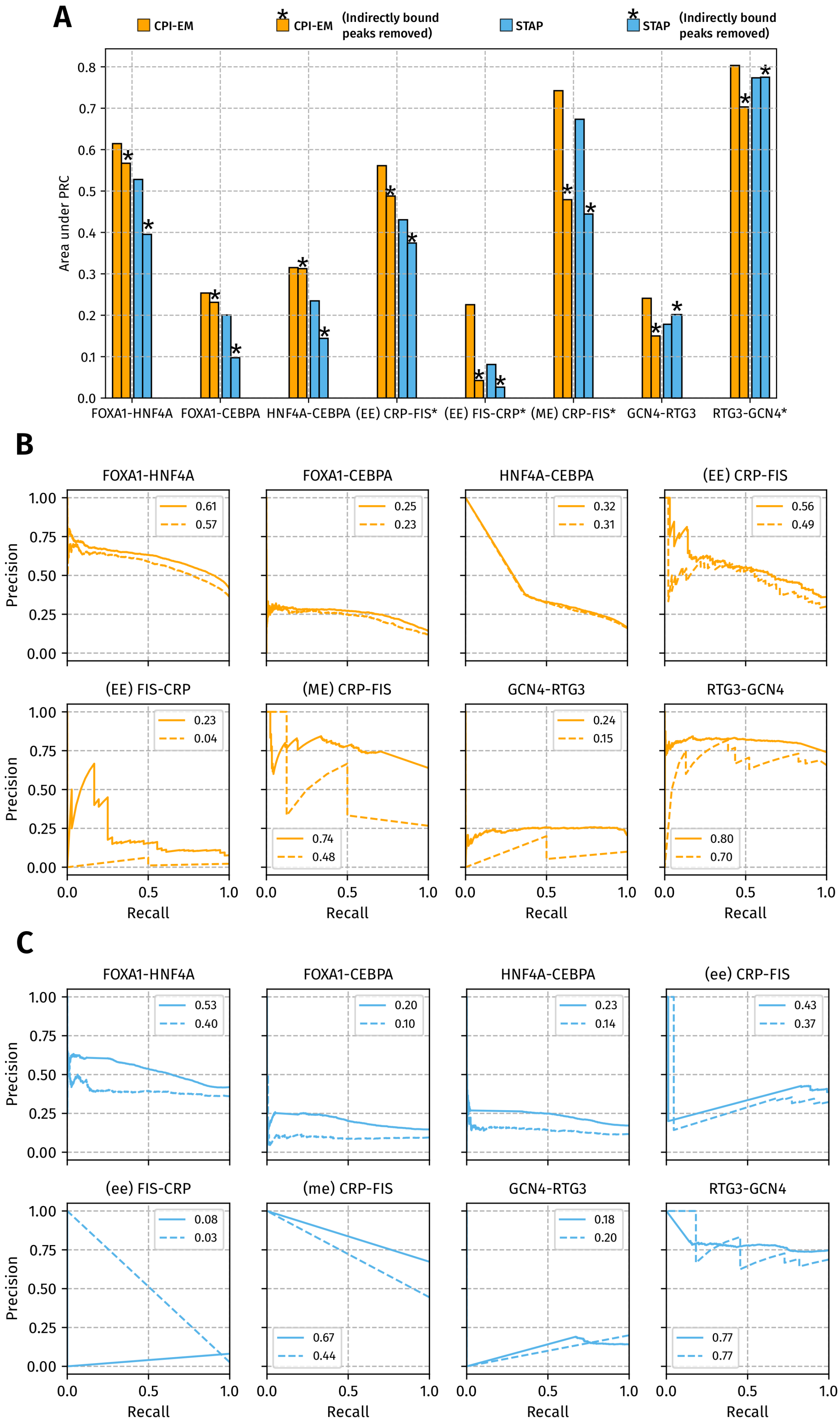

Figure N: (A) The average precision of CPI-EM is higher than that of STAP, even after indirectly bound peaks are removed The average precision of STAP and CPI-EM are in sky blue and orange respectively, while the bars with asterisks above them represent the average precision after indirectly bound peaks are removed from the dataset. The datasets marked with an asterisk (*) are those where STAP was numerically unstable i.e. it did not consistently converge to the same model parameters after multiple runs. **Full precision-recall curves of (B) CPI-EM and (C) STAP for datasets shown in (A)** The precision-recall curves for CPI-EM (orange) and STAP (blue), for datasets where indirectly bound peaks are retained (solid lines) and filtered out (dashed lines). See K Section for the computation of average precision and the precision-recall curves.

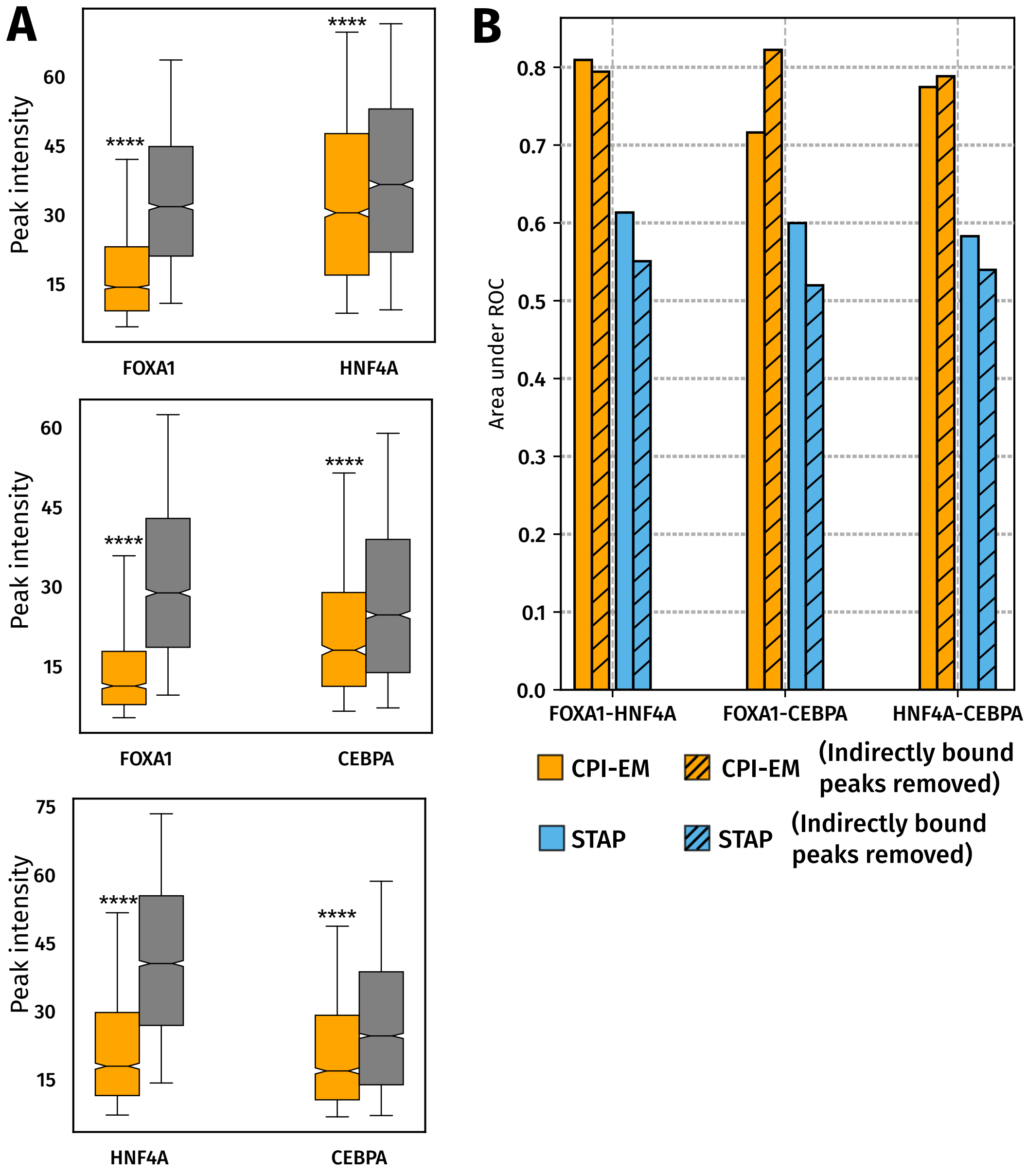

Figure O: Filtering peaks in FOXA1, HNF4A and CEBPA datasets by FDR instead of IDR does not alter trends in peak intensities of cooperatively bound target TF peaks. (A) Peak intensities of cooperatively bound target TFs are still significantly lower than those of non-cooperatively bound target TFs. **** represents p-values of less than 10^{-4} obtained from a Wilcoxon rank-sum test. **(B)** The log-normal variant of CPI-EM outperforms STAP, in terms of the area under their ROC curves, on FOXA1-HNF4A, FOXA1-CEBPA and HNF4A-CEBPA datasets, even after indirectly bound peaks are filtered out.

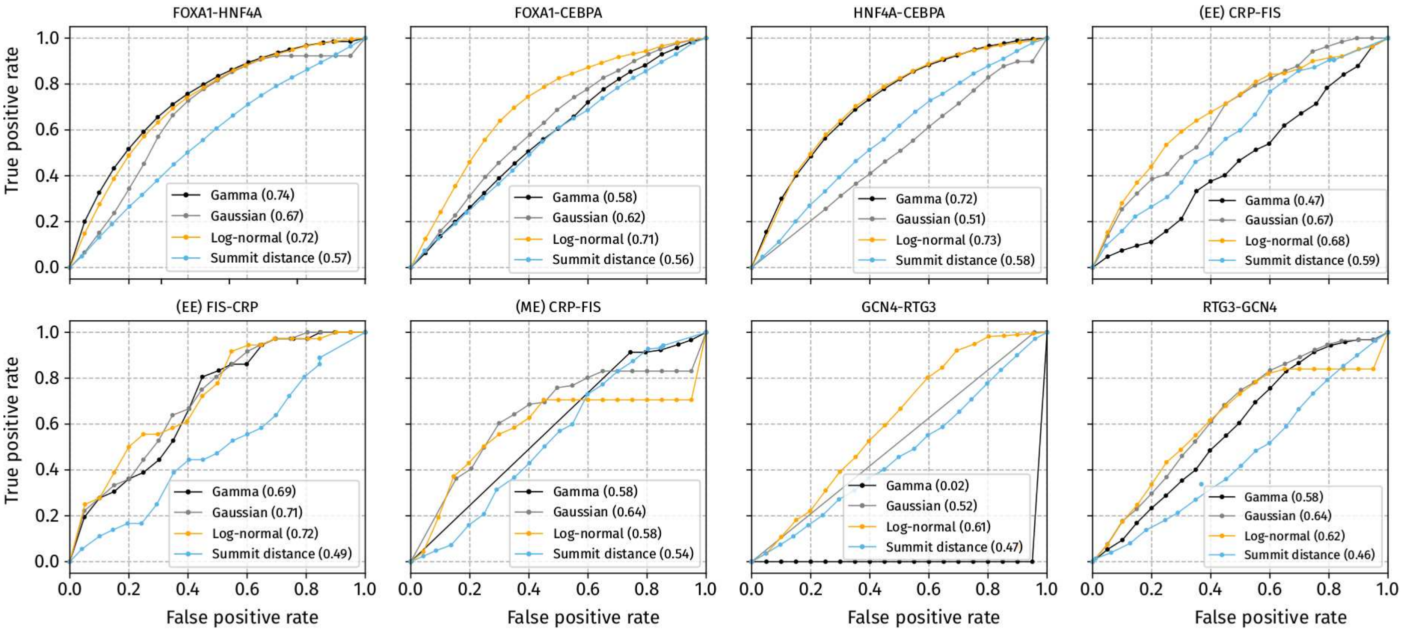

Figure P: ROC curves of runs of the CPI-EM variants (log-normal in orange, Gamma in black, and Gaussian in gray) and peak distance (sky blue) algorithms on datasets in Figure 4 of the main text.

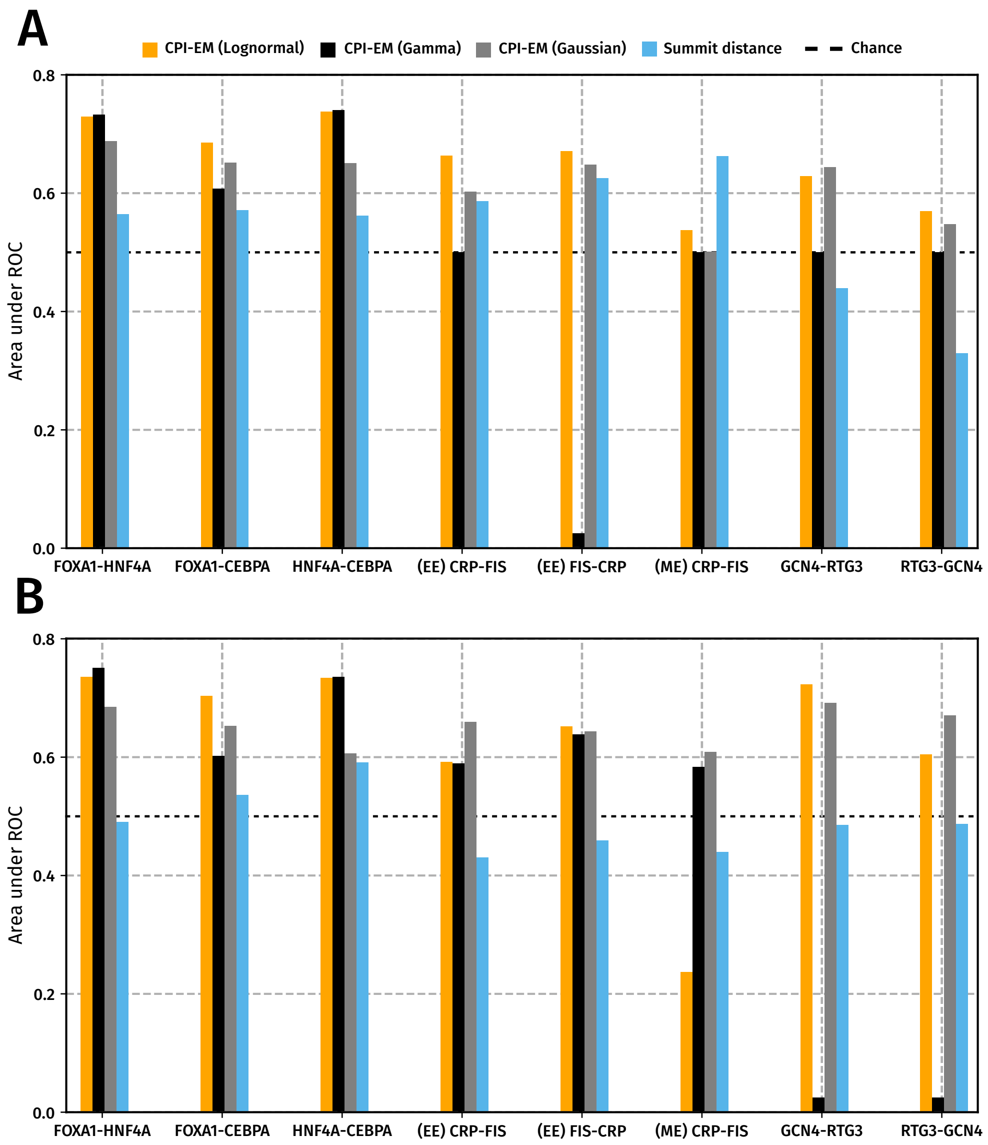

Figure Q: Effect of removing indirectly bound peaks and peak trimming on the performance of each variant of CPI-EM. CPI-EM variants that fit Lognormal, Gamma and Gaussian distributions are represented in orange, black and gray, respectively. The auROC of the peak distance based detector is shown in blue.**(A)** In most datasets, removing indirectly bound peaks has only a minimal impact on the performance of CPI-EM. In the mid-exponential CRP-FIS dataset, however, the Log-normal CPI-EM variant performs worse than chance. **(B)** The trimming of peaks to 50 base pairs on either side of the summit does not affect the performance of CPI-EM in *M. musculus* ChIP-seq data. However, in the mid-exponential CRP-FIS dataset, the Log-normal CPI-EM variant performed worse than chance.

References

[1] Stefflova, K., Thybert, D., Wilson, M. D., Streeter, I., Aleksic, J. , Karagianni, P., Brazma, A., Adams, D. J., Talianidis, I., Marioni, J. C. , et al. Cooperativity and rapid evolution of cobound transcription factors in closely related mammals. *Cell*, 154(3):530–540, 2013.

[2] Gertz, J., Savic, D., Varley, K. E., Partridge, E. C., Safi, A., Jain, P. , Cooper, G. M., Reddy, T. E., Crawford, G. E., and Myers, R. M. Distinct properties of cell-type-specific and shared transcription factor binding sites. *Molecular cell*, 52(1):25–36, 2013.

[3] Verzi, M. P., Shin, H., He, H. H., Sulahian, R., Meyer, C. A., Montgomery, R. K., Fleet, J. C., Brown, M., Liu, X. S., and Shivdasani, R. A. Differentiation-specific histone modifications reveal dynamic chromatin interactions and partners for the intestinal transcription factor CDX2. *Developmental Cell*, 19(5):713–726, 2010.

[4] Li, H. and Durbin, R. Fast and accurate short read alignment with Burrows–Wheeler transform. *Bioinformatics*, 25(14):1754–1760, 2009.

[5] Feng, J., Liu, T., Qin, B., Zhang, Y., and Liu, X. S. Identifying ChIP-seq enrichment using MACS. *Nature Protocols*, 7(9):1728–1740, 2012.

[6] Li, Q., Brown, J. B., Huang, H., and Bickel, P. J. Measuring reproducibility of high-throughput experiments. *The annals of applied statistics*, pages 1752–1779, 2011.

[7] Landt, S. G., Marinov, G. K., Kundaje, A., Kheradpour, P., Pauli, F. , Batzoglou, S., Bernstein, B. E., Bickel, P., Brown, J. B., Cayting, P. , et al. ChIP-seq guidelines and practices of the ENCODE and modENCODE consortia. *Genome Research*, 22(9):1813–1831, 2012.

[8] Spivak, A. T. and Stormo, G. D. Combinatorial cis-regulation in saccharomyces species. *G3: Genes— Genomes— Genetics*, 6(3):653–667, 2016.

[9] Cherry, J. M., Hong, E. L., Amundsen, C., Balakrishnan, R., Binkley, G. , Chan, E. T., Christie, K. R., Costanzo, M. C., Dwight, S. S., Engel, S. R., et al. Saccharomyces genome database: the genomics resource of budding yeast. *Nucleic Acids Research*, page gkr1029, 2011.

[10] Benjamini, Y. and Hochberg, Y. . Controlling the false discovery rate: a practical and powerful approach to multiple testing. *Journal of the Royal Statistical Society. Series B (Methodological)*, pages 289–300, 1995. *Bioinformatics*, 27(24):3423–3424, 2011.

[11] Eric Jones, Travis Oliphant, and Pearu Peterson. {Scipy}: open source scientific tools for {Python}. 2014.

[12] Kinney, J. B. and Atwal, G. S. . Equitability, mutual information, and the maximal information coefficient. *Proceedings of the National Academy of Sciences*, 111(9):3354–3359, 2014.

[13] Shuyang Gao, Greg Ver Steeg, and Aram Galstyan. Efficient estimation of mutual information for strongly dependent variables. In *AISTATS*, 2015.

[14] Dempster, A. P. , Laird, N. M. , and Rubin, D. B. . Maximum likelihood from incomplete data via the em algorithm. *Journal of the royal statistical society. Series B (methodological)*, pages 1–38, 1977.

[15] Durbin, R. , Eddy, S. R. , Krogh, A. , and Mitchison, G. . *Biological sequence analysis: probabilistic models of proteins and nucleic acids.* Cambridge University Press, 1998.

[16] Jeff A. Bilmes and others A gentle tutorial of the EM algorithm and its application to parameter estimation for Gaussian mixture and hidden Markov models *International Computer Science Institute*, 510(4):126, 1998.

[17] Ioannis Kosmidis and Dimitris Karlis. Model-based clustering using copulas with applications. *Statistics and Computing*, 1–21, 2015.

[18] MJD Powell. Direct search algorithms for optimization calculations. *Acta numerica*, pages 287–336, 1998.

[19] Ivan V Kulakovskiy, Ilya E Vorontsov, Ivan S Yevshin, Anastasiia V Soboleva, Artem S Kasianov, Haitham Ashoor, Wail Ba-alawi, Vladimir B Bajic, Yulia A Medvedeva, Fedor A Kolpakov, et al. Hocomoco: expansion and enhancement of the collection of transcription factor binding sites models. *Nucleic acids research*, 44(D1):D116–D125, 2016.

[20] Aaron T Spivak and Gary D Stormo. Scertf: a comprehensive database of benchmarked position weight matrices for saccharomyces species. *Nucleic acids research*, 40(D1):D162–D168, 2011.

[21] Bailey, T. L. , Boden, M. , Buske, F. A. , Frith, M. , Grant, C. E. , Clementi, L. , Ren, J. , Li, W. W. , and Noble, W. S. . Meme suite: tools for motif discovery and searching. *Nucleic acids research*, 37(suppl_2):W202–W208, 2009.

[22] Wang, J. , Zhuang, J. , Iyer, S. , Lin, X. , Whitfield, T. W. , Greven, M. C. , Pierce, B. G. , Dong, X. , Kundaje, A. , Cheng, Y. , et al. Sequence features and chromatin structure around the genomic regions bound by 119 human transcription factors. *Genome research*, 22(9):1798–1812, 2012.

[23] He, X. , Chen, C.-C. , Hong, F. , Fang, F. , Sinha, S. , Ng, H.-H. , and Zhong, S. . A biophysical model for analysis of transcription factor interaction and binding site arrangement from genome-wide binding data. *PloS One*, 4(12):e8155, 2009.

[24] Pei Fen Kuan, Dongjun Chung, Guangjin Pan, James A Thomson, Ron Stewart, and Sündüz Keleş. A statistical framework for the analysis of chip-seq data. *Journal of the American Statistical Association*, 106(495):891–903, 2011.

[25] Thomas Derrien, Jordi Estellé, Santiago Marco Sola, David G Knowles, Emanuele Raineri, Roderic Guigó, and Paolo Ribeca. Fast computation and applications of genome mappability. *PloS one*, 7(1):e30377, 2012.

[26] Shane Neph, M Scott Kuehn, Alex P Reynolds, Eric Haugen, Robert E Thurman, Audra K Johnson, Eric Rynes, Matthew T Maurano, Jeff Vierstra, Sean Thomas, et al. Bedops: high-performance genomic feature operations. *Bioinformatics*, 28(14):1919–1920, 2012.

[27] Fabian Pedregosa, Gaël Varoquaux, Alexandre Gramfort, Vincent Michel, Bertrand Thirion, Olivier Grisel, Mathieu Blondel, Peter Prettenhofer, Ron Weiss, Vincent Dubourg, et al. Scikit-learn: Machine learning in python. *Journal of machine learning research*, 12(Oct):2825–2830, 2011.
